# All-fluorescence white organic light-emitting diodes with record-beating power efficiencies over 130 lm W^‒1^ and small roll-offs

**DOI:** 10.1038/s41467-022-32967-w

**Published:** 2022-09-02

**Authors:** Hao Liu, Yan Fu, Ben Zhong Tang, Zujin Zhao

**Affiliations:** 1 State Key Laboratory of Luminescent Materials and Devices, Guangdong Provincial Key Laboratory of Luminescence from Molecular Aggregates, South China University of Technology, 510640 Guangzhou, China; 2 School of Science and Engineering, Shenzhen Institute of Aggregate Science and Technology, The Chinese University of Hong Kong, Shenzhen, 518172 Guangdong China

**Keywords:** Organic LEDs, Organic LEDs

## Abstract

Improving power efficiency (*PE*) and reducing roll-off are of significant importance for the commercialization of white organic light-emitting diodes (WOLEDs) in consideration of energy conservation. Herein, record-beating *PE* of 130.7 lm W^−1^ and outstanding external quantum efficiency (*EQE*) of 31.1% are achieved in all-fluorescence two-color WOLEDs based on a simple sandwich configuration of emitting layer consisting of sky-blue and orange delayed fluorescence materials. By introducing a red fluorescence dopant, all-fluorescence three-color WOLEDs with high color rendering index are constructed based on an interlayer sensitization configuration, furnishing ultrahigh *PE* of 110.7 lm W^−1^ and *EQE* of 30.8%. More importantly, both two-color and three-color WOLEDs maintain excellent *PE*s at operating luminance with smaller roll-offs than the reported state-of-the-art WOLEDs, and further device optimization realizes outstanding comprehensive performances of low driving voltages, large luminance, high *PE*s and long operational lifetimes. The underlying mechanisms of the impressive device performances are elucidated by host-tuning effect and electron-trapping effect, providing useful guidance for the development of energy-conserving all-fluorescence WOLEDs.

## Abstract (web summary)

High power efficiency and low roll-off values are essential to the commercialization of white organic light-emitting diodes. Here, the authors construct all-fluorescence devices with an orange emitting layer sandwiched between two sky-blue emitting layers, achieving figure-of-merit of 130.7 lm/W.

## Introduction

White organic light-emitting diodes (WOLEDs) are emerging as the kernel for the next-generation display and illumination devices, and substantial efforts have been devoted to functional materials exploration, exciton manipulation and distribution, configuration optimization and fabrication technique development^1–3^. To achieve high electroluminescence (EL) efficiencies, all-phosphorescence systems and fluorescence-phosphorescence hybrid systems are widely studied in reported WOLEDs due to high exciton utilization efficiencies of phosphorescence materials. But the high cost of noble metal-containing phosphorescence materials as well as the lack of robust blue phosphorescence materials impede the widespread commercialization of WOLEDs to a large degree. Purely organic thermally activated delayed fluorescence (TADF) materials can theoretically harness 100% electrogenerated excitons through reverse intersystem crossing (RISC) from the lowest triplet excited (T_1_) state to the lowest singlet excited (S_1_) state based on a small energy gap (Δ*E*_ST_) between S_1_ and T_1_ states^4–7^, and thus have been regarded as the successors of phosphorescence materials. The external quantum efficiency (*EQE*) of monochromatic OLEDs based on TADF emitters have successfully surpassed 30%, and even approached 40% in very recent reports^8–12^. Therefore, certain high-efficiency TADF emitters with competitive prices hold great potentials to replace phosphorescence materials in OLED industry.

On the other hand, TADF materials are generally designed to have highly twisted electron donor-acceptor (D-A) structures to acquire small Δ*E*_ST_s. Such kind of twisted D-A structures can also endow the molecules with ambipolar carrier transport ability, which is favorable for achieving carrier balance and thus high exciton recombination efficiencies in devices. Therefore, TADF materials can behavior as hosts as well as sensitizers for various kinds of luminescent materials to greatly improve device performances because of enhanced efficiencies of exciton production and utilization^13–17^. The conventional fluorescence emitters, which can only harness singlet excitons, particularly benefit from doping in TADF materials, rendering much higher EL efficiencies than those in common host materials. Combining the high color purity and stability of fluorescence emitters, all-fluorescence WOLEDs using TADF materials as both hosts and sensitizers possess superior competitiveness and merits to conventional all-fluorescence WOLEDs based on common fluorescence emitters, bringing about new opportunities for the advancement of WOLEDs^18–24^.

**Fig. 1:**
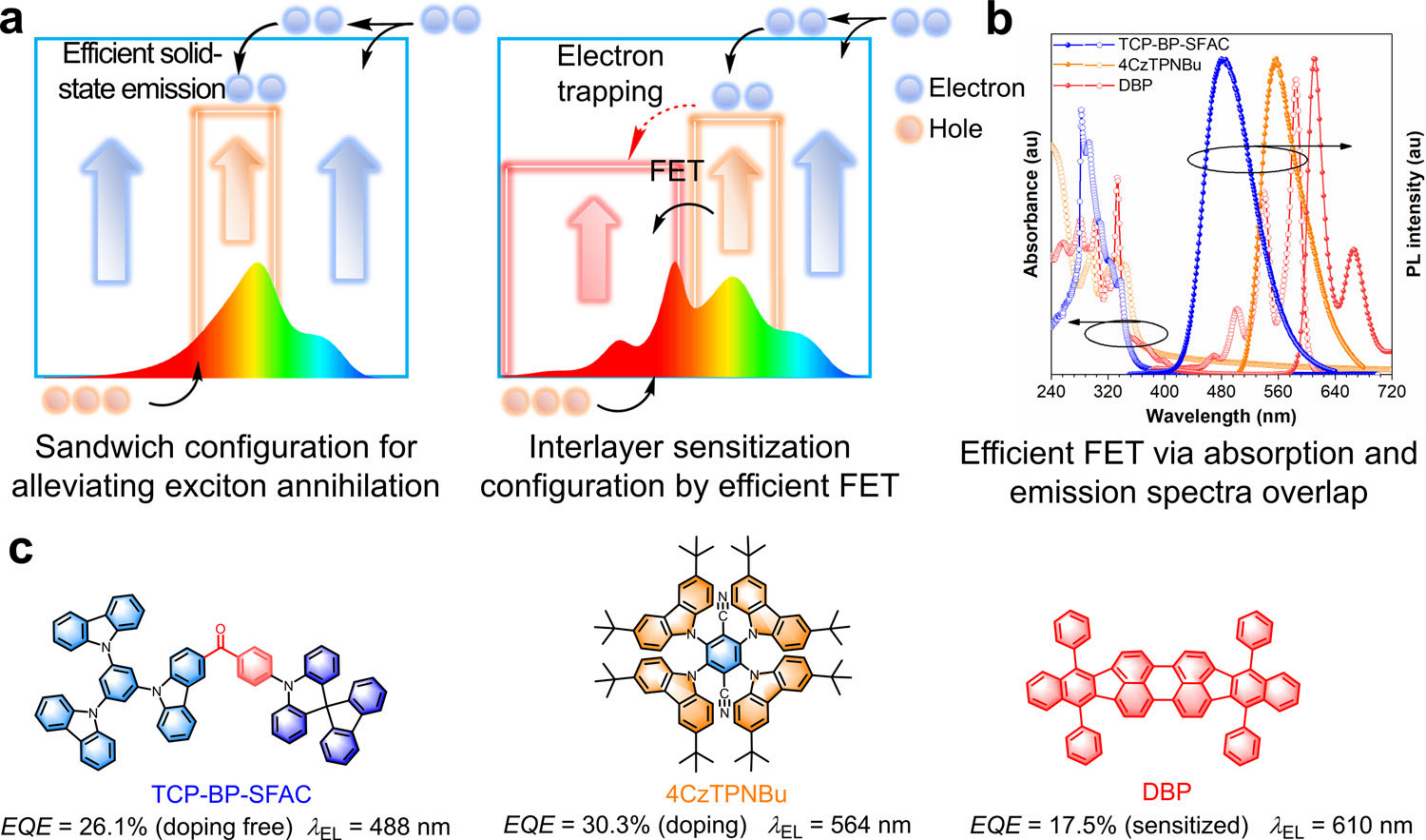
Design principle and materials information of WOLEDs. **a** Schemical mechanisms of sandwich configuration design and interlayer sensitizating system. **b** Absorption and photoluminescence (PL) spectra of sky-blue emitter TCP-BP-SFAC, orange emitter 4CzTPNBu and red emitter DBP applied in all-fluorescence two-color and three-color WOLEDs in this work. **c** Molecular structures, external quantum efficiencies and electroluminescence (EL) peaks of TCP-BP-SFAC, 4CzTPNBu and DBP.

For white light illumination, power conservation is a vital index for WOLEDs, which can be reflected by the power efficiency (*PE*). And the *PE* at operating luminance is a more accurate parameter for the evaluation of WOLED performances. However, most of the efficient WOLEDs still encounter sharp declines in *PE*s at high luminance, and effective approaches to improve *PE*s and reduce *PE* roll-offs are received relatively less attention. The exciton utilization of the emitters and the carrier injection and transport of the devices play critical roles in determining *PE*s, which should be carefully addressed in the design of energy-conserving WOLEDs by properly selecting not only luminescent materials but also host materials. Currently, several new hosts are demonstrated to be promising candidates for WOLEDs. Organic semiconductors generally exhibit asymmetric carrier transport property, in which the mobility of holes is generally much faster than electrons^25^. The n-type hosts with high T_1_ energy levels can erase the barrier of electron injection and balance the transports of electrons and holes, which is conducive to lowering driving voltages of the devices (Fig. 1a)^26^. However, such kind of n-type hosts are difficult to design, and there are only very limited cases of n-type hosts successfully applied in WOLEDs to boost *PE*s. Besides, intermolecular charge transfer host, namely exciplex host, is reported as an effective alternative for solving the power consumption problem, because their ambipolar carrier transport and easy carrier injection can ensure smooth recombination of excitons in emitting layer (EML), rendering low turn-on voltages for WOLEDs^27–30^. Nevertheless, the fabrication complexity involving multielement doping technique often results in failure to acquire desired device performances based on exciplex host. The application of TADF materials as hosts is another promising avenue to enhance *PE*s owing to their narrower energy gaps compared with conventional hosts as well as much better exciton harvesting ability^24^. The ideal multifunctional TADF materials for both hosts and emitters require not only balanced ambipolar carrier transport but also suppressed exciton annihilation and emission quenching in neat films, which is challenging because most reported TADF materials suffer from serious losses of emission and exciton in neat films, and thus have to be doped in other conventional hosts when applied in OLEDs. In consequence, up to now, the successful cases of high-performances WOLEDs based on TADF materials with comparable or superior *PE*s to those of the best phosphorescence materials engaged WOLEDs are extremely rare^30–32^.

In our previous work, a robust sky-blue delayed fluorescence molecule, (4-(spiro[acridine-9,9’-fluoren]−10-yl)phenyl)(9-(3,5-di(carbazol-9-yl)phenyl)carbazol-3-yl)methanone (TCP-BP-SFAC), with balanced ambipolar carrier transport and excellent photoluminescence (PL) efficiency in neat film was created, which provided record-high *EQE*s of 26.1% and 38.6% in nondoped and doped OLEDs, respectively^10^. These intriguing properties enable TCP-BP-SFAC to function as host and emitter simultaneously in WOLEDs. In this contribution, a simple sandwich configuration consisting of an orange EML located between two sky-blue EMLs is proposed to boost the EL efficiencies of all-fluorescence two-color WOLEDs (Fig. 1a), in which the neat film of TCP-BP-SFAC (Fig. 1b, c) is adopted as sky-blue EML, and the doped film of an efficient orange TADF emitter 2,3,5,6-tetrakis(3,6-di-(*tert*-butyl)carbazol-9-yl)−1,4-dicyanobenzene (4CzTPNBu) blended in TCP-BP-SFAC host functions as orange EML. The sandwich configuration effectively alleviates exciton quenching caused by the electron-trapping effect of 4CzTPNBu. Thanks to the balanced ambipolar transportation and high solid-state efficiency of TCP-BP-SFAC, the record-beating *PE* of 130.7 lm W^−1^ is successfully achieved in all-fluorescence two-color warm-white devices. For further improving the color quality, a red EML comprised of a red fluorescence dopant 5,10,15,20-tetraphenylbisbenz[5,6]indeno[1,2,3-cd:1′,2′,3′-lm]perylene (DBP) doped in TCP-BP-SFAC host is introduced, which is sensitized by an efficient interlayer sensitizing configuration via Förster energy transfer (FET) as shown in Fig. 1a. The all-fluorescence three-color WOLEDs achieve nearly the same *EQE*s of 30.8% as two-color devices, as well as high *PE*s over 106 lm W^−1^, which are also pioneering among all-fluorescence three-color WOLEDs reported so far. Importantly, the *PE*s at operating luminance of 100 and 1000 cd m^−2^ are kept at satisfactory levels for both two-color and three-color devices, indicating they are practicable devices for illumination. The in-depth working mechanisms behind these devices are thoroughly studied and the effective design strategy for high-performance all-fluorescence WOLEDs is elucidated in detail, in consideration of the electron-trapping effect and host-tuning effect. Moreover, the operational lifetimes to 50% (*LT*_50_) at the initial luminance of 100 cd m^−2^ reach over 50,000 h, validating the presented design strategies for all-fluorescence WOLEDs are of high potential for practial application.

## Results

### All-fluorescence two-color WOLEDs

**Fig. 2:**
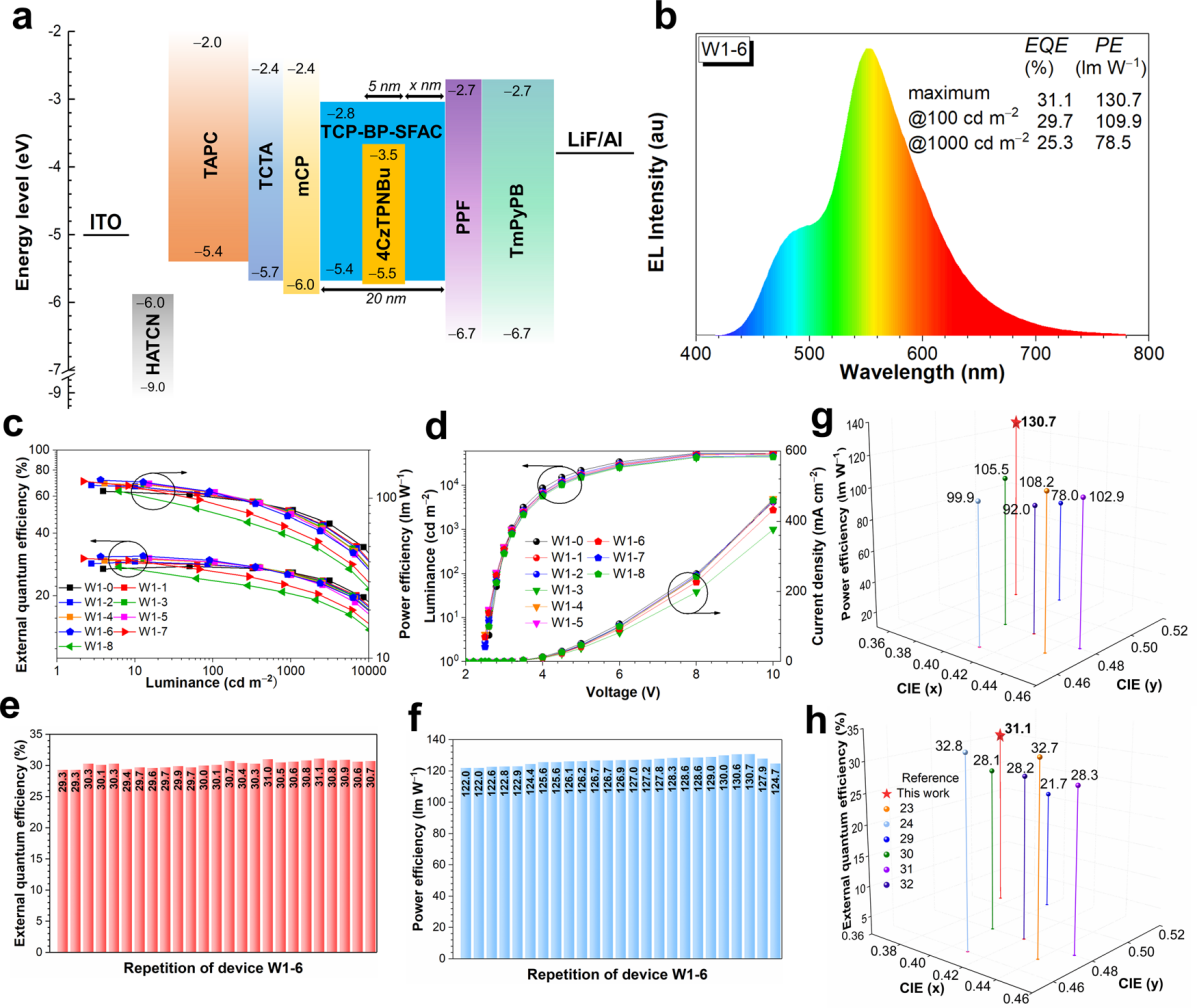
Device data and EL performance of two-color WOLEDs. **a** Device configuration of all-fluorescence two-color WOLEDs. **b** EL spectra of device W1-6 at 1000 cd m^−2^. **c** External quantum efficiency-luminance-power efficiency curves of device W1-0~W1-8. **d** Luminance-voltage-current density curves of device W1-0–W1-8. **e**, **f** Power efficiency and external quantum efficiency of 25 devices based on the configuration of W1-6, respectively. **g**, **h** Power efficiency and external quantum efficiency versus CIE coordinates of representative two-color WOLEDs, respectively. Source data are provided as a  Source Data file.

**Fig. 3:**
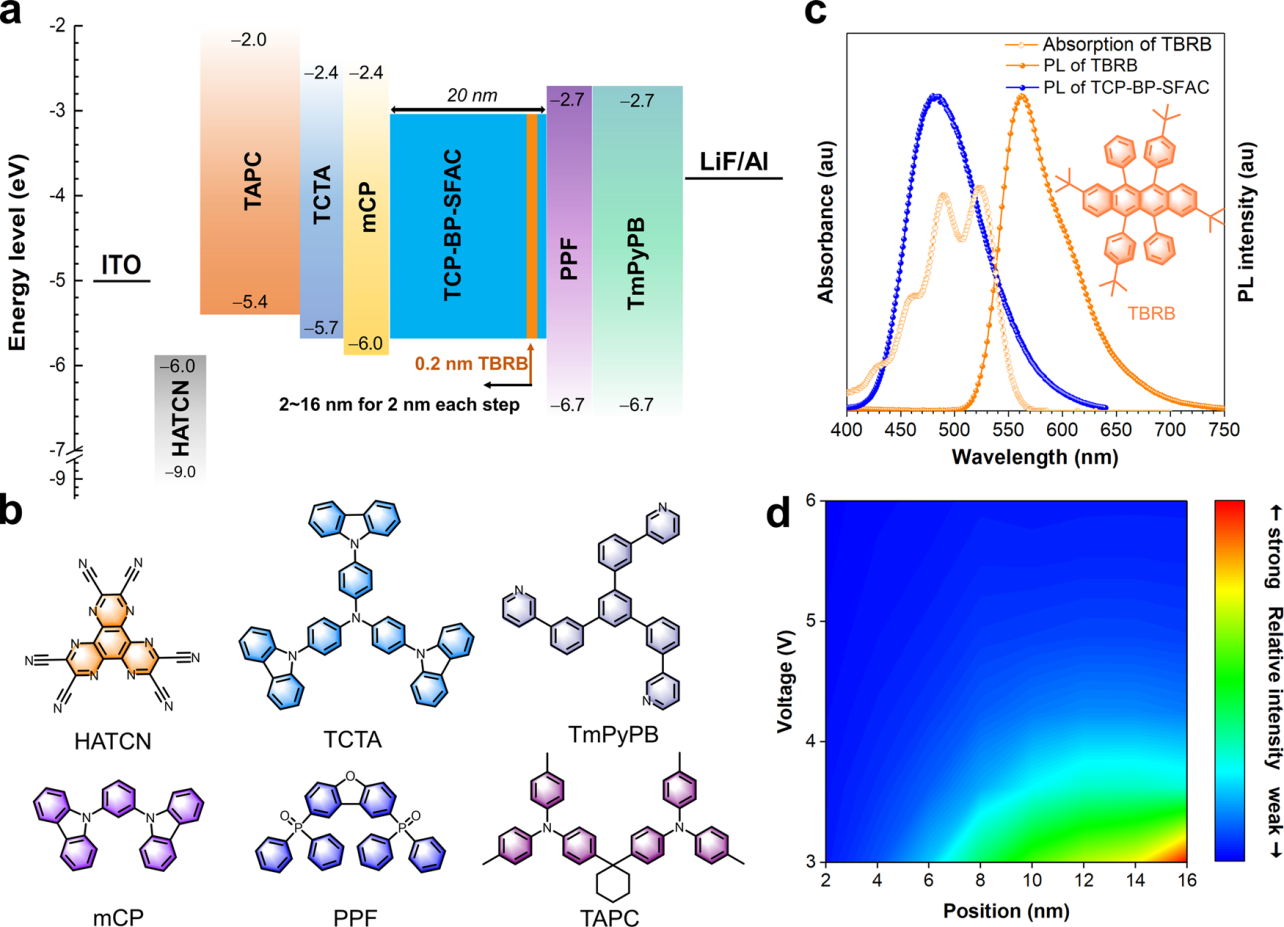
Device data for detecting exciton recombination zone. **a** Device configuration for exciton recombination zone detecting. **b** Molecular structures of the functional materials applied in WOLEDs. **c** Absorption and PL spectra of TBRB in toluene solution and PL spectrum of TCP-BP-SFAC in neat film. **d** Relative intensity of TBRB/TCP-BP-SFAC versus position and voltage. Source data are provided as a Source Data file.

The basic device configuration for all-fluorescence two-color WOLEDs is designed as ITO/HATCN (5 nm)/TAPC (50 nm)/TCTA (5 nm)/mCP (5 nm)/EML/PPF (5 nm)/TmPyPB (40 nm)/LiF (1 nm)/Al, in which indium tin oxide (ITO) is transparent anode, dipyrazino[2,3-f:2′,3′-h]quinoxaline-2,3,6,7,10,11-hexacarbonitrile (HATCN) is hole injection layer, 4,4′-cyclohexylidenebis[*N*,*N-*bis(4-methylphenyl)aniline] (TAPC) is hole-transporting layer, tris[4-(carbazol-9-yl)phenyl]amine (TCTA) functions not only as hole-transporting layer but also electron-blocking layer, 1,3-bis(carbazol-9-yl)benzene (mCP) works as buffer layer between hole-transporting layer and EML in view of its high T_1_ energy level, 2,8-bis(diphenyl-phosphoryl)-dibenzo[b,d]furan (PPF) is exciton-blocking layer, 1,3,5-tri(m-pyrid-3-yl-phenyl)benzene (TmPyPB) is electron-transporting layer, and LiF/Al works as cathode (Supplementary Table 1). A blue-orange-blue sandwich configuration is designed for EML responsible for white EL emission, comprised of TCP-BP-SFAC (15 ‒ *x* nm)/1.5 wt% 4CzTPNBu: TCP-BP-SFAC (5 nm)/TCP-BP-SFAC (*x* nm) (Figs. 2a and 3b), in which *x* = 0–8 by the step of 1 nm, corresponding to devices W1-0–W1-8. The key parameters of devices W1-0–W1-8 are shown in Fig. 2b–d, Supplementary Fig. 1 and Table 1. All the devices can be turned on at low voltages of ~2.6 V and exhibit a low operational voltage of ~3.2 V at luminance of 1000 cd m^−2^. Initially, the EL efficiencies are gradually enhanced along with the increase of *x* and achieve maximum at *x* = 6. Further increasing *x* leads to slight decrease in EL efficiencies. The best device W1-6 radiates intense warm-white light with a maximum luminance (*L*_max_) of 52,690 cd m^−2^, and provides significantly boosted *PE* of 130.7 lm W^−1^ in comparison with those of the reported WOLEDs (<110 lm W^−1^) (Fig. 2g and Supplementary Table 2)^23,24,29–32^. The maximum *EQE* of W1-6 reaches up to 31.1%, which is among the best *EQEs* of the reported WOLEDs (Fig. 2h and Supplementary Table 2). The efficiency distribution of 25 devices with the identical configuration of W1-6 verifies the good reproducibility (Fig. 2e, f). The roll-offs of *PE*s and *EQE*s of all the devices are very small at working luminance. The *EQE* is kept as 29.7% and 25.3% at luminance of 100 cd m^−2^ and 1000 cd m^−2^, respectively. More importantly, outstanding *PE*s are achieved as 108.8 lm W^−1^ and 83.5 lm W^−1^ at luminance of 100 cd m^−2^ and 1000 cd m^−2^, respectively, much better than those of the reported state-of-the-art WOLEDs^23,24,29–32^. The remarkably high *PE*s at working luminance demonstrate the realization of energy conservation of the devices, which makes them more applicable and competitive in practical use (Table 1).

**Table 1:** EL performance of two-color all-fluorescence WOLEDs

Device	Voltage (V)^a^	*CE* (cd A^‒1^)^b^	*PE* (lm W^‒1^)^b^	*EQE* (%)^b^	CIE (x,y)^c^	*L*_max_ (cd m^−2^)^d^
	*V*_on_/100/1000 cd m^−2^	max/100/1000 cd m^−2^	max/100/1000 cd m^−2^	max/100/1000 cd m^−2^		
W1-0	2.6/2.9/3.5	93.7/92.6/75.3	111.0/103.1/67.6	27.7/27.6/24.1	(0.355, 0.510)	36410
W1-1	2.5/2.9/3.2	96.9/94.6/85.0	117.0/105.2/83.5	28.8/28.2/25.8	(0.416, 0.531)	51570
W1-2	2.5/2.8/3.2	95.8/94.7/83.9	120.4/105.9/82.4	29.1/28.3/25.6	(0.415, 0.529)	51670
W1-3	2.5/2.8/3.2	101.4/97.0/82.9	122.5/108.8/81.4	30.0/29.0/25.7	(0.403, 0.522)	48350
W1-4	2.5/2.8/3.2	100.3/96.2/81.8	121.2/108.0/80.3	30.0/29.0/25.5	(0.405, 0.521)	50530
W1-5	2.5/2.8/3.2	101.5/96.7/80.5	122.6/108.5/79.0	30.2/29.1/25.1	(0.396, 0.519)	51310
W1-6	2.5/2.8/3.2	104.2/98.0/78.9	130.7/109.9/78.5	31.1/29.7/25.3	(0.374, 0.505)	52690
W1-7	2.5/2.9/3.2	101.4/85.8/68.5	127.4/95.3/67.2	30.2/26.5/22.7	(0.342, 0.492)	45550
W1-8	2.5/2.9/3.2	91.4/75.4/61.5	110.4/83.8/60.4	27.5/23.9/20.9	(0.323, 0.482)	44010
W2-1	2.5/2.9/3.4	88.0/78.9/63.1	110.7/85.9/63.1	30.9/27.7/22.7	(0.400, 0.483)	47190
W2-2	2.5/2.9/3.4	80.1/75.2/61.7	99.7/83.0/58.2	29.1/27.2/22.4	(0.415, 0.482)	48250
W2-3	2.5/2.9/3.4	73.5/66.2/52.2	92.4/72.8/48.2	29.6/25.9/20.0	(0.415, 0.469)	41380
W3-1	2.4/2.8/3.3	79.5/78.1/69.1	101.7/87.6/66.4	26.0/24.1/21.3	(0.435, 0.514)	99690
W3-2	2.4/3.0/3.5	75.7/72.3/63.2	97.5/75.7/56.8	24.3/23.2/20.4	(0.452, 0.509)	95670
W3-3	2.4/2.8/3.5	62.9/53.8/60.0	82.4/60.4/35.9	22.8/19.2/14.1	(0.461, 0.496)	57230

^a^Operating voltage at 1 (*V*_on_), 100 and 1000 cd m^‒2^*.*

^b^CE/PE/EQE = current efficiency/power efficiency/external quantum efficiency at maximum value, 100 and 1000 cd m^‒2^.

^c^Commission Internationale de l’Eclairage coordinates at 1000 cd m^‒2^.

^d^Maximum luminance.

In order to understand the impressive EL performance, thorough researches on the working mechanism of these devices are conducted. Device W1-0 can be regarded as a dual emissive layer device, and the single carrier devices of the neat film of TCP-BP-SFAC and doped film of 1.5 wt% 4CzTPNBu: TCP-BP-SFAC disclose that 4CzTPNBu has neglectable hole-trapping effect but significant electron-trapping effect in TCP-BP-SFAC host (Supplementary Fig. 2). In that case, the exciton recombination zone in W1-0 will be greatly constrained in a narrow zone in orange EML (Supplementary Fig. 3), which induces unnecessary energy loss due to triplet-triplet annihilation and triplet-singlet annihilation^33^. By increasing the thickness of TCP-BP-SFAC layer adjacent to PPF layer, the excitons can partially recombine at TCP-BP-SFAC during electron shifting to 4CzTPNBu. Thereby, the excitons can be distributed in a dispersed manner in a wide zone, which alleviates exciton quenching caused by accumulation as depicted in Fig. 1a. The appropriate frontier molecular orbitals of TCP-BP-SFAC also bring about low turn-on voltages comparable with those of WOLEDs based on exciplex hosts^29^. The EL efficiencies tend to decline by continuously increasing the thickness of TCP-BP-SFAC layer (*x* = 7 and 8), because the orange EML gets too far away to harvest sufficient excitons and many excitons are recombined in TCP-BP-SFAC layer as depicted in the EL spectra (Supplementary Fig. 1). The EL efficiency of TCP-BP-SFAC in neat film is lower than that of 4CzTPNBu in doped film, which is probably responsible for the efficiency decline in devices W1-7 and W1-8.

Moreover, an orange fluorescence emitter TBRB is used as detector to study the FET in device (Fig. 3a). The absorption spectrum of TBRB is finely overlapped with the PL spectrum of TCP-BP-SFAC (Fig. 3c), indicating sufficient FET can happen from TCP-BP-SFAC to TBRB. An ultrathin (0.2 nm) layer of TBRB neat film is inserted into the TCP-BP-SFAC layer at varied positions, and the relative emission intensity of TBRB/TCP-BP-SFAC is recorded to draw the relative intensity-position-voltage graph (Fig. 3d). It is found that the exciton recombination zone tends to locate near the hole-transporting part at low voltages, but is more evenly distributed over the entire EML at high voltages. Therefore, it is deduced that the electrons can arrive at 4CzTPNBu at low voltages with partial recombination in TCP-BP-SFAC layer, and shift toward another TCP-BP-SFAC layer through 4CzTPNBu when the operating voltage is increased, leading to enhanced blue component in white light. So, the thickness of TCP-BP-SFAC layer in two-color WOLEDs should be controlled within an appropriate range. A thick layer (a large *x*) of TCP-BP-SFAC adjacent to PPF layer will weaken the contribution of 4CzTPNBu, but a thin layer (a small *x*) may result in exciton accumulation and annihilation at 4CzTPNBu. The function of TCP-BP-SFAC layer adjacent to mCP layer in the sandwich configuration is also investigated. To verify the necessity of this TCP-BP-SFAC layer, double-layer devices WS1 with an EML of 1.5 wt% 4CzTPNBu: TCP-BP-SFAC (5 nm)/TCP-BP-SFAC (6 nm) and WS2 with an EML of 1.5 wt% 4CzTPNBu: TCP-BP-SFAC (14 nm)/TCP-BP-SFAC (6 nm) are fabricated for comparison (Supplementary Fig. 4 and Supplementary Tables 1 and 3). WS1 shows obviously decreased *PE* of 90.7 lm W^−1^ and *EQE* of 23.2% in comparison with W1-6. But with a thicker layer of 1.5 wt% 4CzTPNBu: TCP-BP-SFAC (14 nm), WS2 can provide comparable *PE* of 124.9 lm W^−1^ and *EQE* of 30.1% to those of W1-6. This result discloses this TCP-BP-SFAC layer can serve as a buffer layer to alleviate exciton annihilation, in addition to balance carrier transport in the device.

### All-fluorescence three-color WOLEDs

**Fig. 4:**
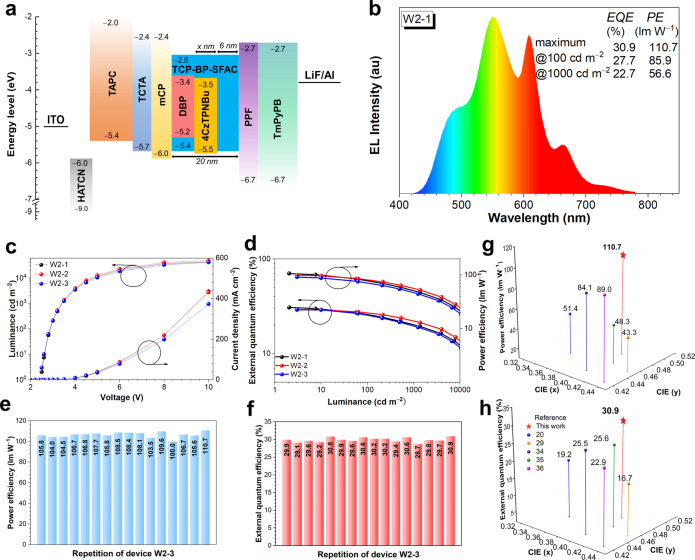
Device data and EL performance of three-color WOLEDs. **a** Device configuration of all-fluorescence three-color WOLEDs. **b** EL spectra of W2-1 at 1000 cd m^−2^. **c** External quantum efficiency-luminance-power efficiency curves of W2-1–W2-3. **d** Luminance-voltage-current density curves of W2-1–W2-3. **e**, **f** Power efficiency and external quantum efficiency of 16 devices based on the configuration of W2-1, respectively. **g**, **h** Power efficiency and external quantum efficiency versus CIE coordinates of representative three-color WOLEDs, respectively. Source data are provided as a Source Data file.

The light sources with high color rendering index (CRI) have diverse operating occasions including indoor-illumination, backlight, mirror lamp, decorative lighting, etc. The conventional fluorescence dopants have advantages of high color quality and stability, which are favorable to increase CRI of WOLEDs, but often encounter the problem of inferior EL efficiencies due to low exciton utilization. The interlayer sensitization system was previously demonstrated as an effective approach to sensitize conventional fluorescence dopants with reduced exciton loss for WOLEDs. Herein, a novel design of interlayer sensitization configuration on the basis of above sandwich configuration of two-color devices is proposed for three-color WOLEDs to have a higher CRI. Three-color white EMLs consisting of 1 wt% DBP: TCP-BP-SFAC (14 ‒ *x* nm)/1.5 wt% 4CzTPNBu: TCP-BP-SFAC (*x* nm)/TCP-BP-SFAC (6 nm) are prepared, in which *x* is 5, 4 and 3 for devices W2-1, W2-2 and W2-3, respectively (Fig. 4a). Different from the sandwich configuration, one of the TCP-BP-SFAC layers is doped with red fluorescence DBP to form a red EML to improve CRI. This red EML is envisioned to be sensitized by adjacent orange EML through FET to obtain high EL efficiencies. The detailed EL performances of W2-1–W2-3 are displayed in Fig. 4b–d, Supplementary Fig. 5 and Table 1. The CRI is successfully increased from 65 of W2-1 to 71 of W2-3, which is close to those of normal lighting devices. Inspiringly, W2-1 achieves remarkable EL performances with a *PE* of 110.7 lm W^−1^ and an *EQE* of 30.8%. The repeated devices of W2-1 have also been fabricated (Fig. 4e, f), which confirms the good reproducibility of the device. W2-2 and W2-3 also exhibit excellent EL performances, with *PE*s and *EQE*s of 99.7 lm W^−1^ and 29.1%, and 92.4 lm W^−1^ and 29.6%, respectively. Like two-color WOLEDs, these three-color WOLEDs also hold high-efficiency stability. The *PE*s of W2-1, W2-2 and W2-3 are maintained at 80.6, 83.0 and 72.8 lm W^−1^ at 100 cd m^−2^, and 52.6, 58.2 and 48.2 lm W^−1^ at 1000 cd m^−2^, respectively, disclosing these devices are amongst the best three-color WOLEDs (Fig. 4g, h and Supplementary Table 4)^20,29,34–36^.

To decipher the sensitization process, a supplementary device WS3 with an EML of 1 wt% DBP: mCP (5 nm)/1.5 wt% 4CzTPNBu: TCP-BP-SFAC (14 nm)/TCP-BP-SFAC (6 nm) is fabricated, which is coincident with the reported interlayer sensitization design (Supplementary Fig. 4 and Supplementary Tables 1 and 3)^37^. WS3 shows excellent *PE* and *EQE* of 126.8 lm W^−1^ and 30.6%, respectively, but the EL spectrum of WS3 is barely changed in comparison with that of W1-6, in which the characteristic peak of DBP cannot be observed. The primary cause for the failed sensitization is the large span between the recombination zones of 4CzTPNBu and DBP, due to severe electron-trapping of 4CzTPNBu as illustrated in Supplementary Fig. 3. Another supplementary device WS4 with an EML consisting of a single doped film of 1 wt% DBP: TCP-BP-SFAC (20 nm) is fabricated (Supplementary Table 1). WS4 has an *EQE* of only 4.9%, close to the *EQE* limit of the conventional fluorescence devices, indicating TCP-BP-SFAC is not a suitable sensitizer for DBP and causes severe exciton loss, namely DBP cannot be sensitized by TCP-BP-SFAC host. Besides, additional interlayers of mCP or TCP-BP-SFAC are inserted in device W2-1 between orange 4CzTPNBu and red DBP layers, and the supporting devices of W2-1-a with a mCP layer of 3 nm, W2-1-b with a mCP layer of 6 nm, W2-1-c with a TCP-BP-SFAC layer of 3 nm, and W2-1-d with a TCP-BP-SFAC layer of 6 nm are prepared (Supplementary Fig. 5). The characteristic red emission of DBP is obviously weakened in W2-1-a and W2-1-c, and nearly disappeared in W2-1-b and W2-1-d, revealing the presence of the interlayer, particularly with a larger thickness, causes significant decrease in emission intensity of DBP. These results also disclose that both mCP and TCP-BP-SFAC have failed in sensitizing DBP, which provide evidences to support that 4CzTPNBu dominates the sensitization of DBP.

**Fig. 5:**
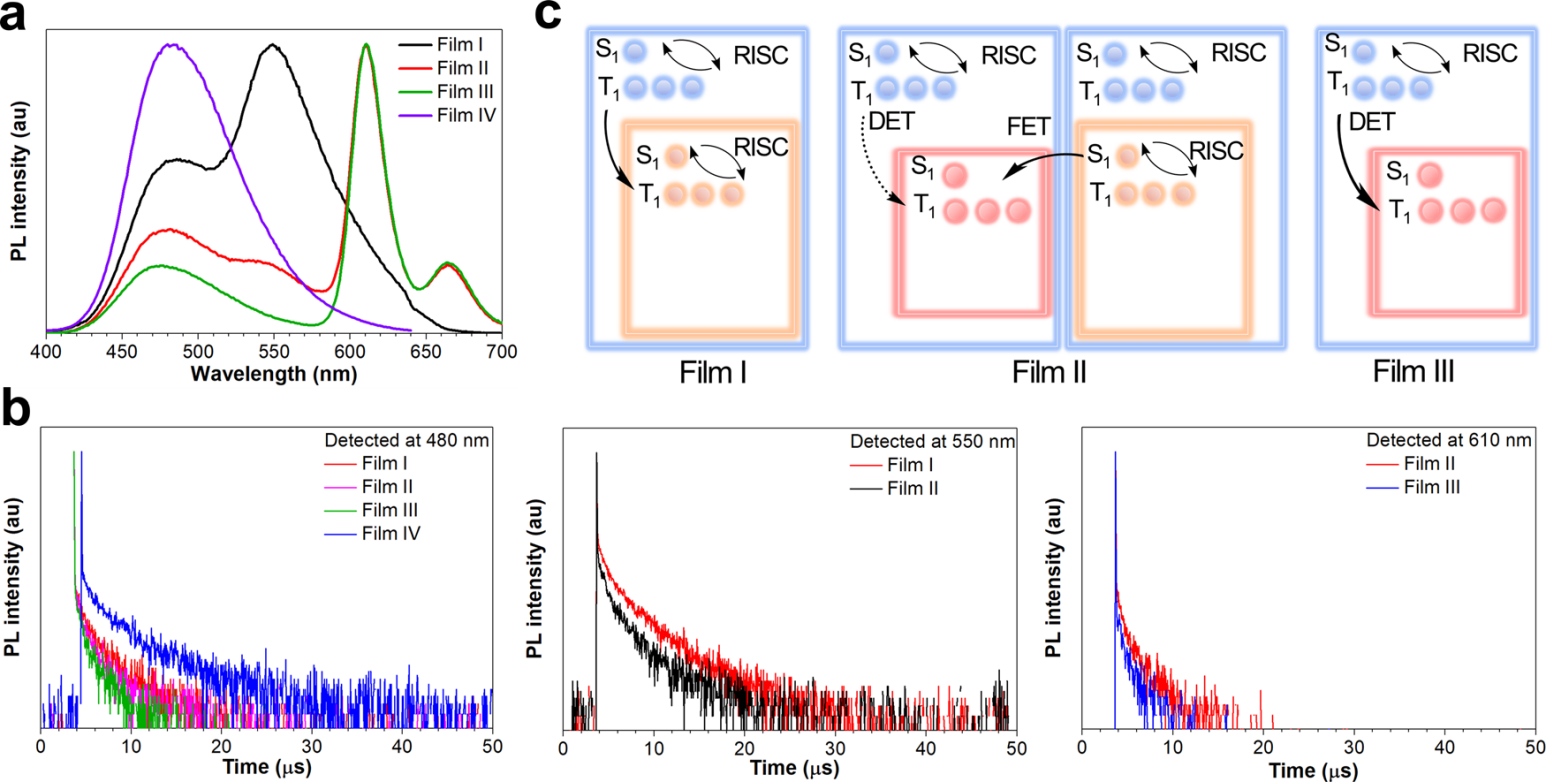
Mechanism study of sensitizing process. **a** PL spectra of films I~IV. **b** Transient PL decay curves of films I–IV. **c** Mechanism illustration of exciton behaviors in films I–III, in which blue, orange and red rectangles represent TCP-BP-SFAC, 4CzTPNBu and DBP, respectively. Source data are provided as a Source Data file.

Further, four films of 1.5 wt% 4CzTPNBu: TCP-BP-SFAC (15 nm) (film I), 1 wt% DBP: TCP-BP-SFAC (9 nm)/1.5 wt% 4CzTPNBu: TCP-BP-SFAC (5 nm) (film II), 1 wt% DBP: TCP-BP-SFAC (15 nm) (film III), and TCP-BP-SFAC (15 nm) (film IV) are prepared to study the sensitization process. As illustrated in Fig. 5a, the PL peaks of TCP-BP-SFAC, 4CzTPNBu and DBP are located at 480, 550 and 610 nm, respectively, which are consistent with their intrinsic PL properties. The transient PL decay curves detected at these PL peaks reveal that film IV has the longest decay lifetime at 480 nm (Fig. 5b and Supplementary Table 5). The mechanism of exciton behaviors is depicted in Fig. 5c. The TADF dopant 4CzTPNBu has lower lying S_1_ and T_1_ states than TCP-BP-SFAC, which can induce exciton diffusion toward 4CzTPNBu, thus leading to reduced delayed lifetime of TCP-BP-SFAC in film I. Film II contains red and orange layers with the identical configuration to that of white EML in W2-1. The delayed lifetime of film II at 550 nm is shortened relative to that of film I. Meanwhile, conventional fluorescence dopant DBP has a considerable decay lifetime of 1.55 μs in film II, much longer than that in toluene solution (3.4 ns) (Supplementary Fig. 8f), evidencing efficient FET from 4CzTPNBu to DBP. This is also supported by their overlapped absorption and PL spectra (Fig. 1b). In film III, TCP-BP-SFAC exhibits the shortest delayed lifetime, and DBP has the reduced delayed lifetime as well, reflecting non-radiative process dominates in film III due to the Dexter energy transfer between T_1_ states from TCP-BP-SFAC to DBP. These findings further manifest that the FET occurs between DBP and 4CzTPNBu but not between DBP and TCP-BP-SFAC.

### Host-tuning effect in WOLEDs

**Fig. 6:**
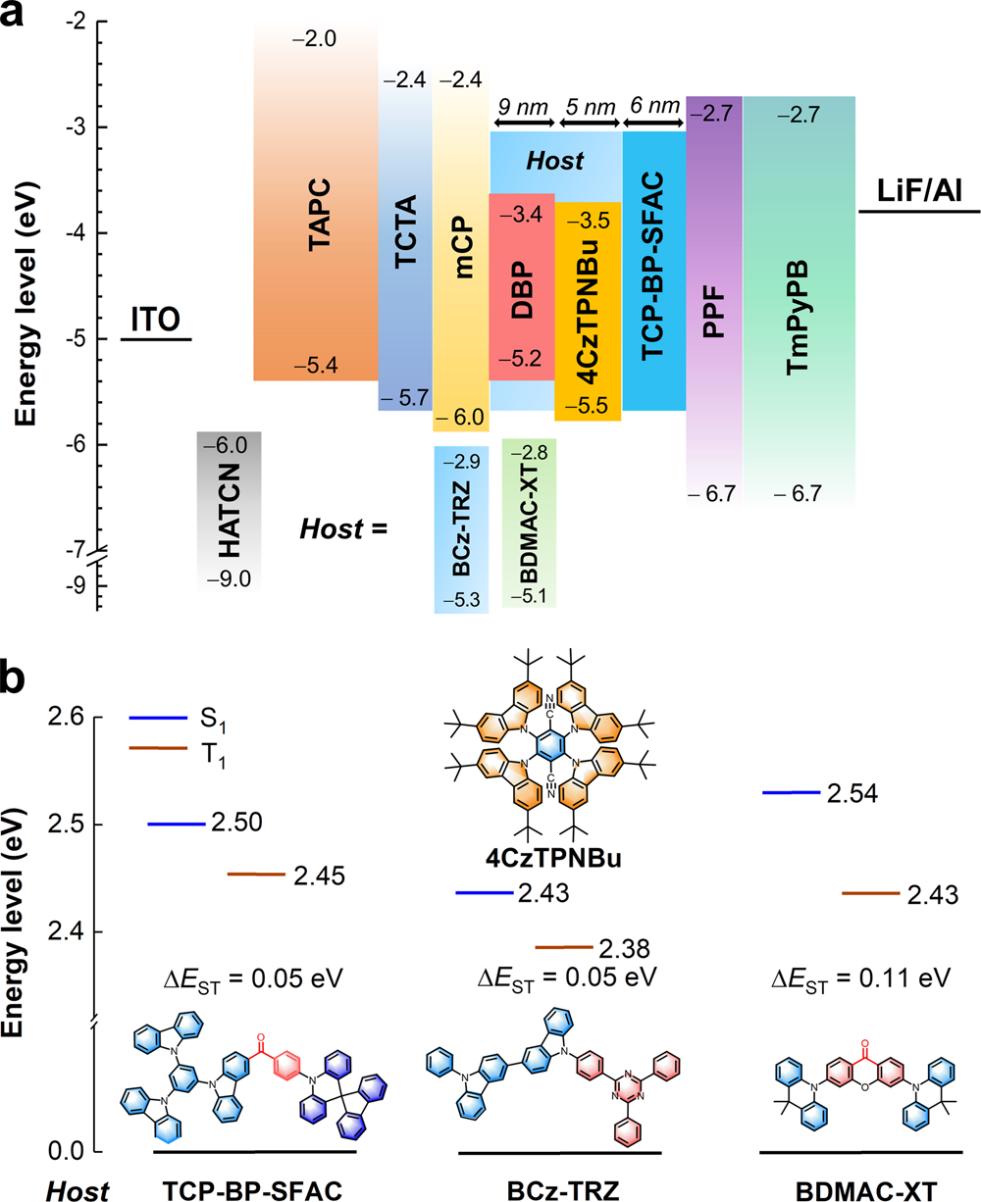
Device data and energy levels for host-tuning effect study. **a** Device configurations of devices WS5–WS7. **b** Singlet and triplet energy levels of 1.5 wt% 4CzTPNBu in TCP-BP-SFAC, BCz-TRZ and BDMAC-XT hosts. Source data are provided as a Source Data file.

The photophysical results and supporting devices validate efficient FET from 4CzTPNBu to DBP through the interlayer sensitization configuration, finally producing impressive EL performance of W2-1–W2-3. Therefore, the EL performance of 4CzTPNBu in TCP-BP-SFAC plays an important role in determining the performance of WOLEDs. To have a better understanding of the influence of host on the EL performance of 4CzTPNBu, two typical TADF materials with similar emission colors, 3,6-bis(9,9-dimethylacridin-10-yl)-xanthen-9-one (BDMAC-XT)^38^ and 9-(4-(4,6-diphenyl-1,3,5-triazin-2-yl)phenyl)−9′-phenyl-9H,9′H-3,3′-bicarbazole (BCz-TRZ)^39^, are selected as control hosts for comparison with TCP-BP-SFAC. Monochromatic devices Y1–Y3 of 4CzTPNBu are fabricated, using TCP-BP-SFAC, BCz-TRZ and BDMAC-XT as hosts, respectively (Supplementary Table 1). Device Y1 exhibits the best *EQE* of 26.1%, device Y3 has a close *EQE* of 25.8% to that of Y1, but Y2 only gives a lower *EQE* of 22.6% (Supplementary Table 3, Supplementary Fig. 6). To figure out the cause, film V (1.5 wt% 4CzTPNBu: BCz-TRZ (15 nm)) and film VI (1.5 wt% 4CzTPNBu: BDMAC-XT (15 nm)) are constructed to compare with film I (1.5 wt% 4CzTPNBu: TCP-BP-SFAC (15 nm)). The Δ*E*_ST_s are 0.05, 0.05 and 0.11 eV for 4CzTPNBu in films I, V and VI, respectively, estimated from the onsets of fluorescence and phosphorescence spectra at 77 K (Supplementary Fig. 8). According to the previous study, the varied Δ*E*_ST_s of a TADF emitter in different hosts are actually associated with the different polarities of the hosts and the valence shell transition types of S_1_ and T_1_ states of the TADF emitter^10^. The natural transition orbital analysis discloses that the S_1_ and T_1_ states of 4CzTPNBu are dominated by π‒π* transition (Supplementary Fig. 9), without the participation of n-orbital^10,40^. To verify this theoretical calculation result, the fluorescence and phosphorescence spectra associated with S_1_ and T_1_ state, respectively, are measured in low-polar toluene and high-polar dichloromethane solutions. Both of the fluorescence and phosphorescence spectra exhibit obvious red-shifts in dichloromethane relative to toluene, which is in accordance with the nature of π‒π* transition^41^, rather than n‒π* transition that generally exhibits the opposite phenomenon^10^. Moreover, the molecular polarity index (MPI) values of S_1_ and T_1_ states of TCP-BP-SFAC, BCz-TRZ and BDMAC-XT are different (Supplementary Table 6). Therefore, the energy levels of the S_1_ and T_1_ states of 4CzTPNBu can be altered in these hosts, leading to different Δ*E*_ST_s (Fig. 6b). So, the RISC and thus delayed fluorescence property are tuned effectively by these hosts (Supplementary Table 5). On the other hand, in comparison with BCz-TRZ, TCP-BP-SFAC and BDMAC-XT have a prominent advantage of effectively preventing excitons from annihilation, as evidenced by their much better EL performances in neat films. To this point, TCP-BP-SFAC can reconcile both the reduction of Δ*E*_ST_ for 4CzTPNBu and the suppression of exciton annihilation, finally resulting in the best performance of monochromatic device Y1.

Moreover, to further test the host impact on the performance of WOLEDs, control devices WS5 and WS6 are fabricated, in which the TCP-BP-SFAC host for 4CzTPNBu and DBP in W2-1 are substituted with BDMAC-XT (device WS5) and BCz-TRZ (device WS6) (Fig. 6a, Supplementary Table 1 and Supplementary Fig. 10). WS5 and WS6 have maximum *EQEs* of 23.2% and 23.9%, respectively, inferior to that of W2-1 (Supplementary Fig. 7 and Supplementary Table 3). The single carrier devices of BDMAC-XT and BCz-TRZ films with or without 4CzTPNBu dopant are fabricated and studied (Supplementary Fig. 2). The electron-trapping effect from 4CzTPNBu is found to be still significant in BDMAC-XT, but is less effective in BCz-TRZ, probably due to its lower lying LUMO. WS6 has the device efficiency coincident with the monochromatic device Y2, while WS5 has lowered efficiency relative to device Y3. The HOMO energy level of BDMAC-XT is shallower than that of TCP-BP-SFAC, which may result in exciton annihilation at the interface of BDMAC-XT/TCP-BP-SFAC, and even the shift of exciton recombination zone toward DBP. Moreover, the control device WS7 containing an EML of BDMAC-XT (9 nm)/1.5 wt% 4CzTPNBu: TCP-BP-SFAC (5 nm)/TCP-BP-SFAC (6 nm) is fabricated. WS7 has a maximum *EQE* of 28.2%, lower than that of W1-6, further confirming that the excitons are partially recombined in BDMAC-XT. These results virtually uncover the energy levels of hosts can significantly influence exciton recombination and distribution zone, and thus can be adopted to effectively tune device performance. So, in these WOLEDs, TCP-BP-SFAC functions as not only sky-blue emitter but also host, and its proper polarity, matched energy levels and ability to suppress exciton annihilation contribute greatly to the outstanding device performances.

### Operational stability optimization

**Fig. 7:**
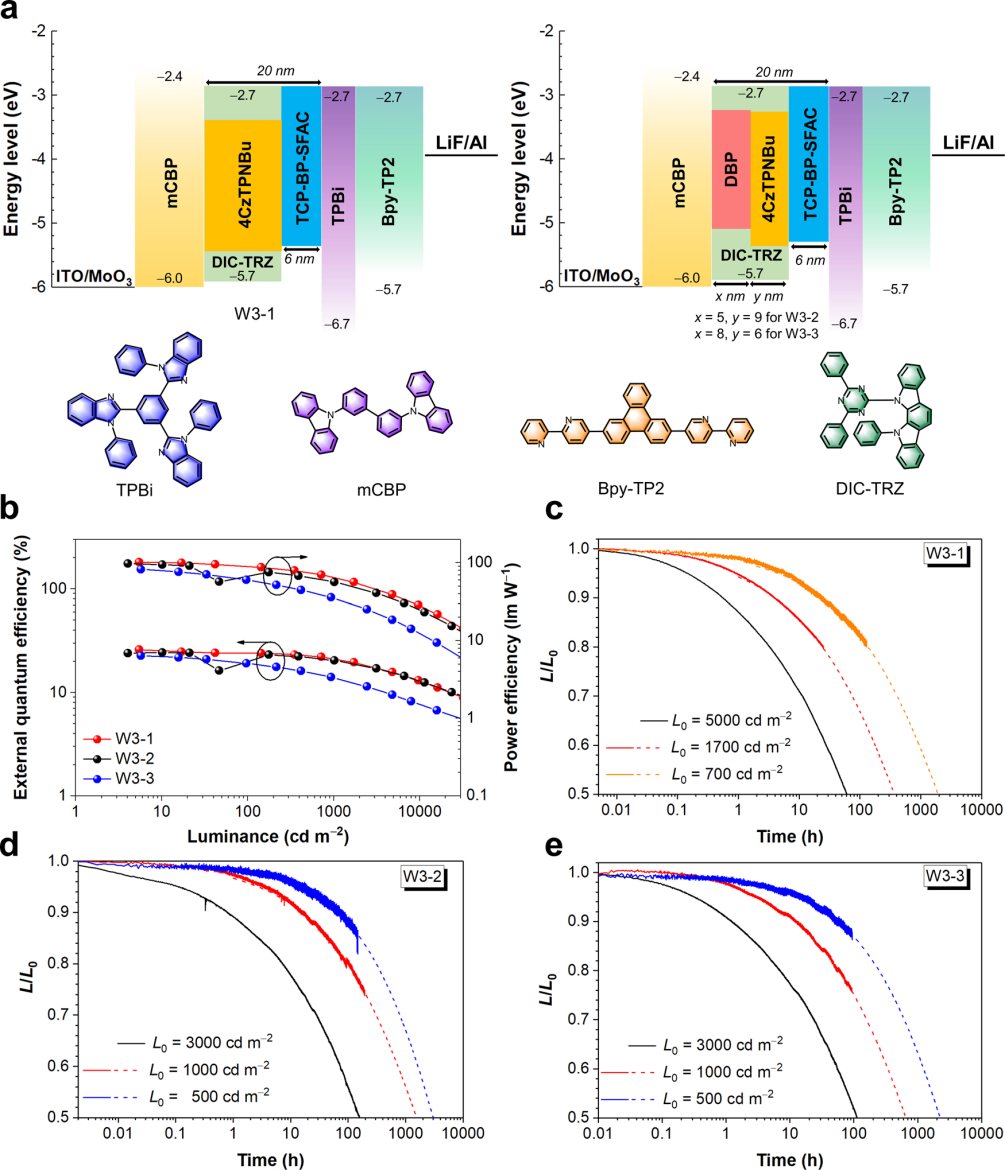
Device data and EL performance of the devices with improved operational lifetimes. **a** Device configurations of W3-1 (left), and W3-2 and W3-3 (right) with molecular structures of additional functional materials. **b** External quantum efficiency-luminance-power efficiency curves of devices W3-1–W3-3. **c**–**e** Operational lifetimes of devices W3-1, W3-3 and W3-3, respectively. The dashed lines are fitted from the tested values (solid lines). Source data are provided as a Source Data file.

Device lifetimes are crucial parameters to evaluate practical application potential of WOLEDs, which are greatly influence by not only device configuration design but also the stability of the functional layers. The functional layers for the highest device efficiency are actually not always suitable for achieving the best stability of the device^42–44^. To gain outstanding comprehensive performances by giving considerations to EL efficiencies and lifetimes of WOLEDs, preliminary device configuration optimization is conducted with commercially available functional materials at hand. Two-color device W3-1 with a configuration of ITO/MoO_3_ (6 nm)/mCBP (40 nm)/1.5 wt% 4CzTPNBu: DIC-TRZ (14 nm)/20 wt% BCz-TRZ: TCP-BP-SFAC (6 nm)/TPBi (5 nm)/Bpy-TP2 (40 nm)/LiF (1 nm)/Al is prepared (Fig. 7a), in which MoO_3_ is adopted as hole injection layer, 3,3′-di(9H-carbazol-9-yl)-1,1′-biphenyl (mCBP) serves as hole-transporting layer, 2,4-diphenyl-6-bis(12-phenylindolo)[2,3-a]carbazole-11-yl)-1,3,5-triazine (DIC-TRZ) is an ambipolar host with good stability^45^, 2,2′,2″-(1,3,5-benzinetriyl)-tris(1-phenyl-1-*H*-benzimidazole) (TPBi) is employed as hole-blocking layer, 2,7-bis(2,2′-bipyridine-5-yl)triphenylene (Bpy-TP2) works as electron-transporting layer with high stability^46^. The TCP-BP-SFAC layer is doped with BCz-TRZ to alleviate exciton quenching, and carrier injection is still kept intact under this design. The perfect carrier transport balance of DIC-TRZ and TCP-BP- SFAC^10^ can play a constructive role in retarding device degradation. This device configuration actually has been greatly simplified to reduce the Joule heat generation, which is conducive to prolonging device lifetimes as well^47^. W3-1 acquires warm lights and the maximum *PE* and *EQE* of 101.7 lm W^−1^ and 26.0%, respectively (Fig. 7b, Supplementary Fig. 10 and Table 1). The *PE*s are kept at 87.6 and 66.4 lm W^−1^ at 100 and 1000 cd m^−2^, respectively, indicative of small roll-offs. The *L*_max_ reaches 99690 cd m^−2^, implying the great potential to achieve high operational stability. The values of *LT*_50_ at initial luminance of 5000, 1700 and 700 cd m^−2^ are 61, 368 and 1927 h, respectively (Fig. 7c). At initial luminance of 100 cd m^−2^, *LT*_50_ is fitted to be 56337 h. Moreover, three-color devices W3-2 and W3-3 with configurations of ITO/MoO_3_ (6 nm)/mCBP (40 nm)/1 wt% DBP: DIC-TRZ (5 nm)/1.5 wt% 4CzTPNBu: DIC-TRZ (9 nm)/20 wt% BCz-TRZ: TCP-BP-SFAC (6 nm)/TPBi (5 nm)/Bpy-TP2 (40 nm)/LiF (1 nm)/Al and ITO/MoO_3_ (6 nm)/mCBP (40 nm)/1 wt% DBP: DIC-TRZ (8 nm)/1.5 wt% 4CzTPNBu: DIC-TRZ (6 nm)/20 wt% BCz-TRZ: TCP-BP-SFAC (6 nm)/TPBi (5 nm)/Bpy-TP2 (40 nm)/LiF (1 nm)/Al are fabricated. W3-2 and W3-3 possess the maximum *PE*s of 97.5 and 82.4 lm W^−1^, and *EQE*s of 24.3% and 22.8%, respectively. The *PE*s of W3-2 and W3-3 are decreased moderately at operating luminance (Supplementary Fig. 10 and Table 1). The *L*_max_s of W3-2 and W3-3 are 95670 and 57230 cd m^−2^, respectively. The *LT*_50_ values at initial luminance of 3000, 1000 and 500 cd m^−2^ are recorded as 158, 1548 and 3029 h for W3-2, and 113, 652 and 2242 h for W3-3 (Fig. 7d, e). The fitted values of *LT*_50_ at initial luminance 100 cd m^−2^ are 55335 and 31462 h for W3-2 and W3-3, respectively. These results validate the good operational stability of these devices, which is favored for practical application. In these optimized WOLEDs, some functional materials are changed and the device configurations are simplified, but the basic design strategy of the EMLs remains intact. These outstanding comprehensive device performances unambiguously further proves the applicability of our design strategy for all-fluorescence WOLEDs.

## Discussion

In summary, all-fluorescence two-color WOLEDs with a sandwich configuration consisting of an orange EML of delayed fluorescence 4CzTPNBu doped in delayed fluorescence TCP-BP-SFAC host located between two sky-blue EMLs of TCP-BP-SFAC neat films is proposed. Owing to the outstanding EL efficiency, small carrier injection barrier and balanced ambipolar carrier transport of TCP-BP-SFAC in neat film, the turn-on voltages of the devices are successfully lowered, and ultrahigh *PE*s and *EQE*s of up to 130.7 lm W^−1^ and 31.1% are achieved. The sandwich configuration effectively alleviates exciton quenching caused by the electron-trapping effect of 4CzTPNBu. Based on the best performed two-color device configurations, a red EML of red fluorescence DBP doped in TCP-BP-SFAC host is introduced to improve the color quality, which is sensitized by the adjacent orange EML via an interlayer sensitization configuration. In such device configuration, the electron-trapping effect of 4CzTPNBu can exert positive effect of preventing excitons from recombining at DBP dopant, and the FET from 4CzTPNBu greatly enhances the red emission efficiency of DBP. The generated all-fluorescence three-color WOLEDs attain remarkable *PE*s and *EQE*s of up to 110.7 lm W^−1^ and 30.8%. More importantly, both two-color and three-color WOLEDs not only acquire record-beating *PE*s but also enjoy superb efficiency stability, with much smaller *PE* roll-offs at operational luminance than those of the state-of-the-art WOLEDs. Finally, on the basis of most efficient two-color and three-color device configurations, further device optimization furnishes outstanding comprehensive performances of low driving voltages, large luminance, high EL efficiencies and long operational lifetimes for WOLEDs. The mechanism investigation demonstrates that, in addition to mathched HOMO and LUMO energy levels, suitable host-tuning and electron-trapping effects and the ability to suppress exciton annihilation of the host contribute significantly for realizing these high-performance WOLEDs. The design strategy for constructing highly efficient sensitization system presented in this work holds great practical potentials for the advancement of energy-conserving WOLEDs.

## Methods

### Materials and instruments

The compounds HATCN, TAPC, TCTA, mCP, mCBP, PPF, TmPyPB, TPBi, Bpy-TP2, 4CzTPNBu, BCz-TRZ and DIC-TRZ were purchased from commercial sources. TCP-BP-SFAC and BDAMC-XT were synthesized according the published methods^10,38^. PL spectra were recorded by Horiba Fluoromax-4 spectrofluorometer, and UV-visible absorption spectra were taken from a Shimadzu UV-2600 spectrophotometer. Transient PL decay curves and fluorescence lifetimes were measured under nitrogen atmosphere at room temperature in a Quantaurus-Tau fluorescence lifetime measurement system (C11367-03, Hamamatsu Photonics Co., Japan).

### Device fabrication and characterization

Glass substrates precoated with 90 nm ITO with resistance of 15–20 Ω per square were successively cleaned with acetone, isopropanol, detergent and deionized water in ultrasonic bath. After that, all substrates were dried in an oven maintaining at 70 °C. In order to improve the hole-injection process in WOLED devices, all substrates were treated by O_2_ plasma for 10 min. All of the WOLED devices were fabricated by vacuum deposition method under a pressure <5 × 10^‒4^ Pa in the Fangsheng OMV-FS 450 vacuum deposition system. Organic materials, LiF and Al were deposited at the rates of 1–2 Å s^‒1^, 0.1 Å s^‒1^ and 5 Å s^‒1^, respectively. All the devices had their luminance-voltage-current density characteristics and EL spectra measured in a PhotoResearch PR670 spectroradiometer, along with a Keithley 2400 Source Meter. The effective emitting area of the devices was 9 mm^2^, determined by the overlap between anode and cathode. All the characterizations were conducted at room temperature in ambient conditions without any encapsulation except for device lifetime measurement, as soon as the devices were fabricated.

### Theoretical calculation

The frontier molecular orbitals of TCP-BP-SFAC, 4CzTPNBu and DBP were optimized using the density functional theory (DFT) method with PBE0-D3 functional at the basis set level of 6-31 G (d, p), and the natural transition orbitals of 4CzTPNBu were optimized using the time-dependent DFT method with PBE0-D3 functional at the basis set level of 6-31 G (d, p). The above calculations were performed using Gaussian16 package. The MPI values, and natural transition orbital analysis were analyzed with Multiwfn.

## Supplementary information

Supplementary Information

## Data Availability

The source data underlying Figs. 2b‒h, 3c, d, 4b‒h, 5a, b, 6b, 7b‒e and Supplementary Figs. 1a, b, 2, 4a‒h, 5a, c, 6a‒d, 7a‒d, 8a‒f, 10a‒c are provided with this paper as a Source Data file. All the other data supporting the findings of this study within the artile and its supplementary information are available from the corresponding author upon request. Source data are provided with this paper.

## Source data

Source Data
