# Supplementary material for: All-fluorescence white organic light-emitting diodes with record-beating power efficiencies over 130 lm W‒1 and small roll-offs
Source: Nat Commun. 2022 Sep 2;13:5154. doi: 10.1038/s41467-022-32967-w (PMC9440051; doi:10.1038/s41467-022-32967-w)
Supplement: Supplementary file 1 — Supplementary Information [file 41467_2022_32967_MOESM1_ESM.pdf]

SUPPLEMENTARY INFORMATION

All-fluorescence white organic light-emitting diodes with record-beating power efficiencies over 130 lm W^{-1} and small roll-offs

Hao Liu,[1] Yan Fu,[1] Ben Zhong Tang[2] and Zujin Zhao[1,*]

[1] State Key Laboratory of Luminescent Materials and Devices, Guangdong Provincial Key Laboratory of Luminescence from Molecular Aggregates, South China University of Technology, Guangzhou, 510640, China.

[2] School of Science and Engineering, Shenzhen Institute of Aggregate Science and Technology, The Chinese University of Hong Kong, Shenzhen, Guangdong 518172, China.

* Corresponding Author: Zujin Zhao (email address: mszjzhao@scut.edu.cn)

Table of contents

Supplementary Figures 3
Supplementary Fig. 1 3
Supplementary Fig. 2 3
Supplementary Fig. 3 4
Supplementary Fig. 4 4
Supplementary Fig. 5 5
Supplementary Fig. 6 5
Supplementary Fig. 7 6
Supplementary Fig. 8 6
Supplementary Fig. 9 7
Supplementary Fig. 10 7
Supplementary Tables 8
Supplementary Table 1 8
Supplementary Table 2 9
Supplementary Table 3 10
Supplementary Table 4 10
Supplementary Table 5 10
Supplementary Table 6 11
Supplementary References 11

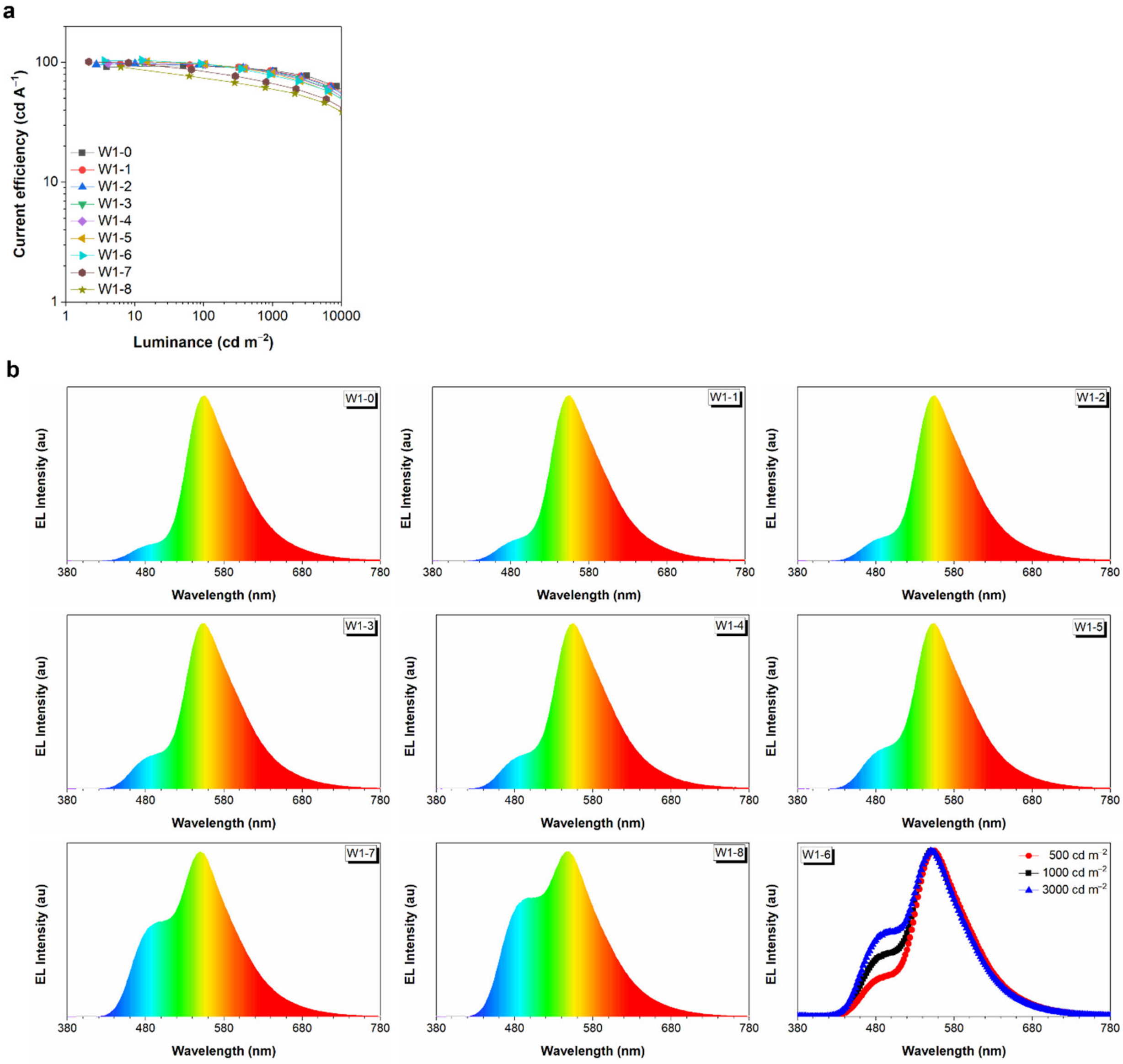

Supplementary Figure 1. a Current efficiency-luminance curves and **b** EL spectra of devices W1-1~W1-6, and W1-8~W1-9 at ~1000 cd m^{-2}, and EL spectra of device W1-6 at 500, 1000 and 3000 cd m^{-2}. Source data are provided as a Source Data file.

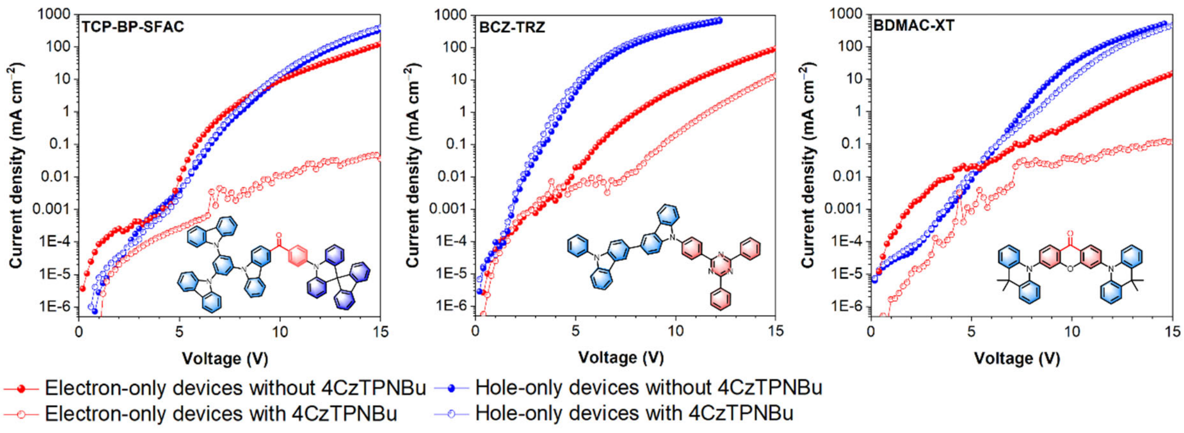

Supplementary Figure 2. Current density-voltage curves of single carrier devices. The configurations are ITO/TAPC

(50 nm)/EMLs (20 nm)/TAPC (40 nm)/Al (hole-only devices) and ITO/TmPyPB (50 nm)/EMLs (20 nm)/TmPyPB (40 nm)/LiF (1 nm)/Al (electron-only devices), in which the EMLs are the neat films of TCP-BP-SFAC, BCz-TRZ and BDMAC-XT, or their doped films containing 1.5 wt% 4CzTPNBu. Source data are provided as a Source Data file.

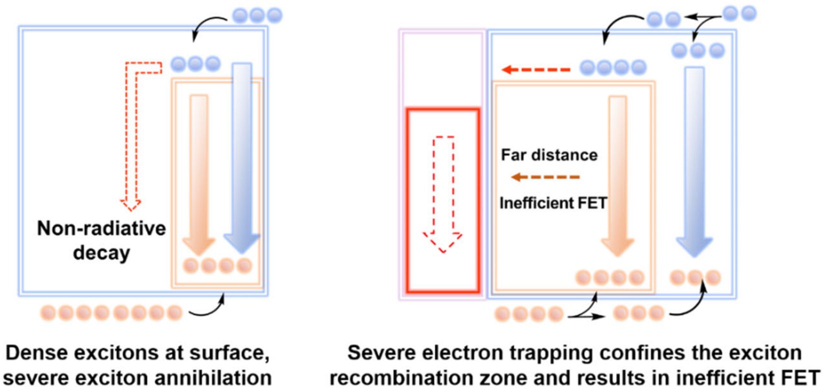

Supplementary Figure 3. Mechanism illustration of exciton behaviors in devices W1-0 (left) and WS3 (right).

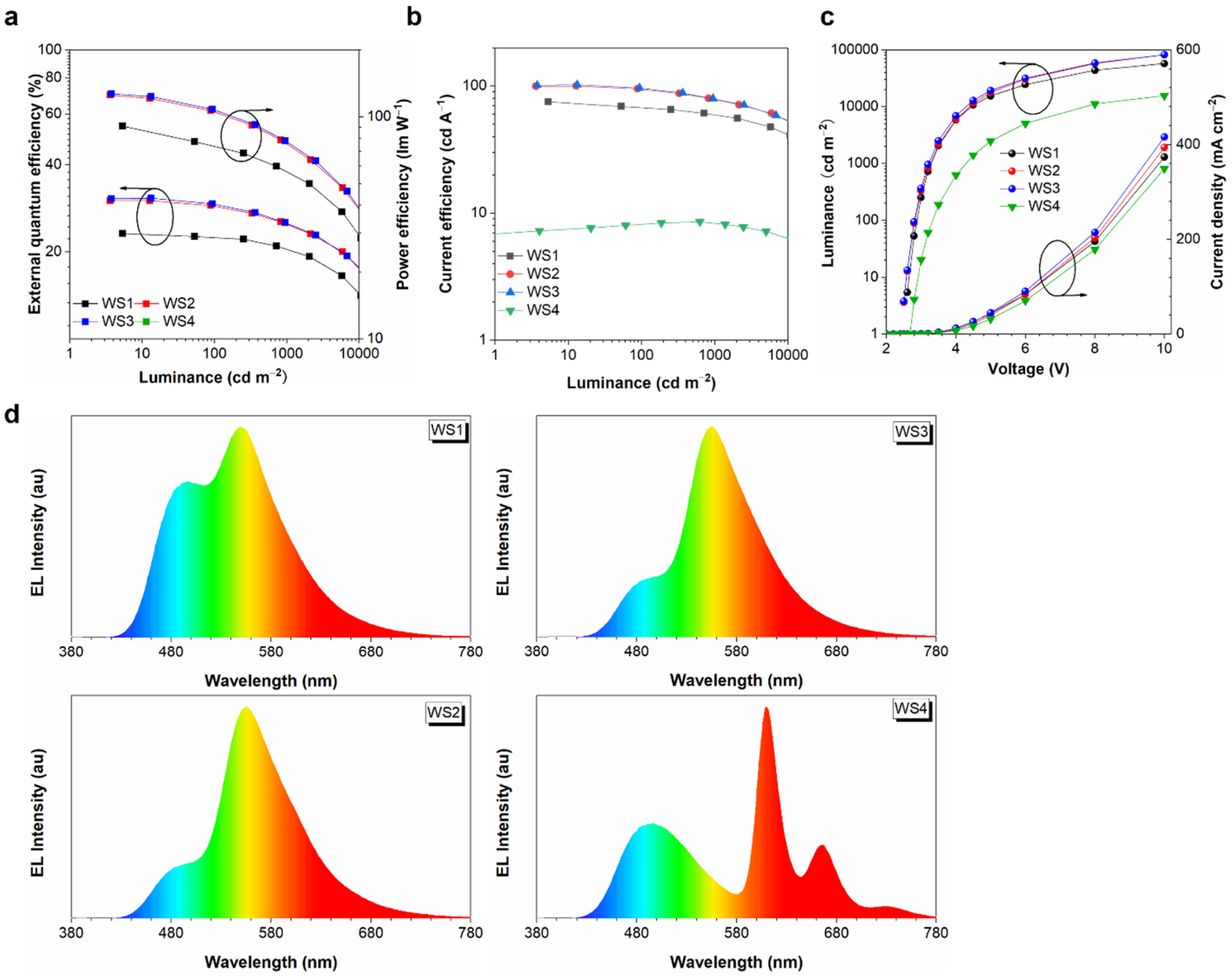

Supplementary Figure 4. a External quantum efficiency-luminance-power efficiency, **b** current efficiency-luminance and **c** luminance-voltage-current density curves of devices WS1~WS4. **d** EL spectra of devices WS1~WS4 at ~1000 cd m^{-2}. Source data are provided as a Source Data file.

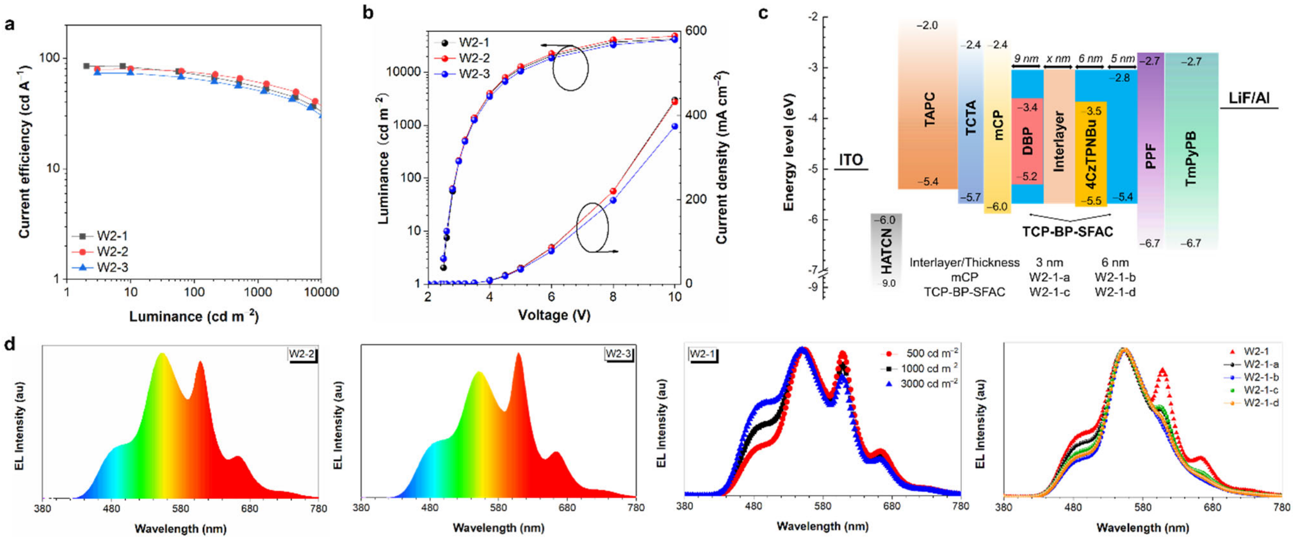

Supplementary Figure 5. a Current efficiency-luminance curves of devices W2-1~ W2-3. **b** Device configuration of W2-1-a~W2-1-d. **c** EL spectra of devices W2-2 and W2-3 at 1000 cd m^{-2}, EL spectra of device W2-1 at 500, 1000 and 3000 cd m^{-2}, and EL spectra of W2-1-a~W2-1-d at ~1000 cd m^{-2}. Source data are provided as a Source Data file.

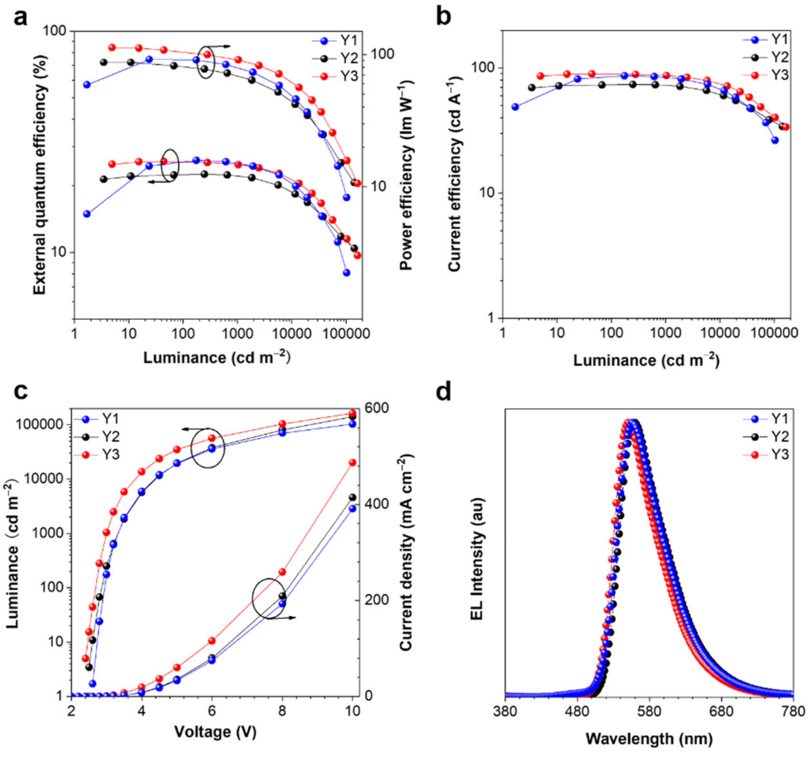

Supplementary Figure 6. a External quantum efficiency-luminance-power efficiency, **b** current efficiency-luminance, **c** luminance-voltage-current density curves and **d** EL spectra of devices Y1~Y3 at ~1000 cd m^{-2}. Source data are provided as a Source Data file.

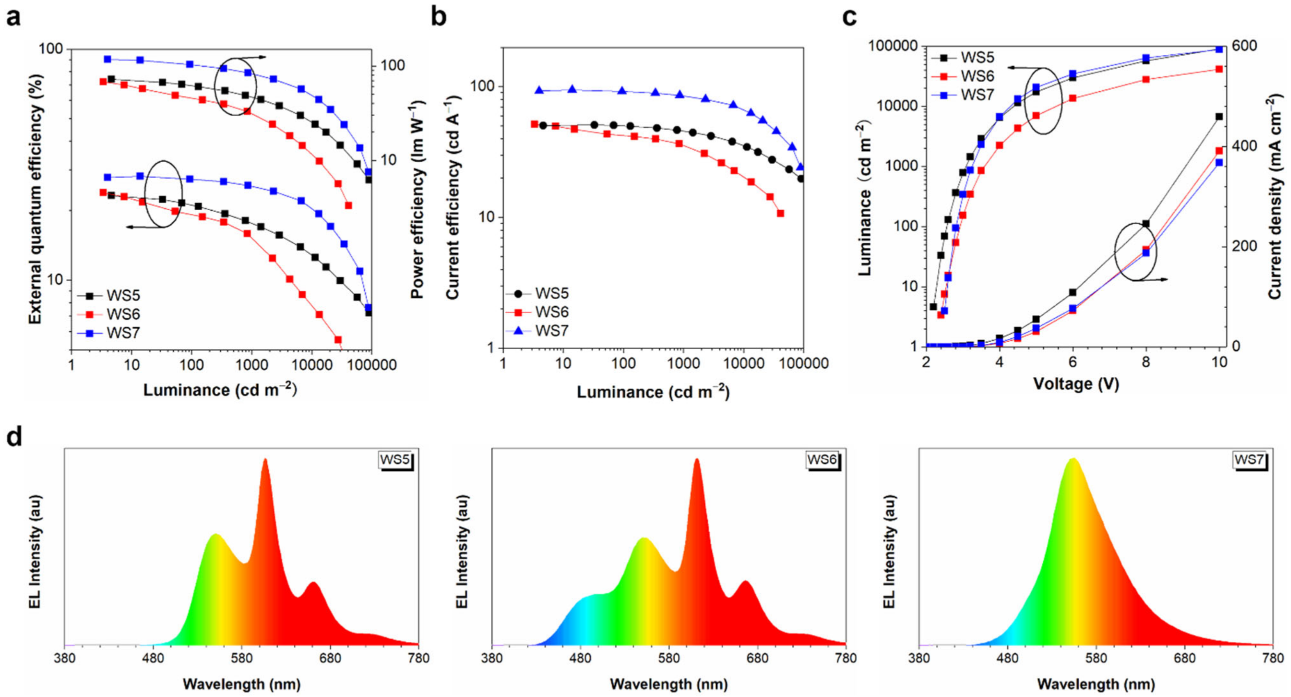

Supplementary Figure 7. **a** External quantum efficiency-luminance-power efficiency, **b** current efficiency-luminance, **c** luminance-voltage-current density curves and **d** EL spectra of devices WS5~WS7 at ~1000 cd m^{-2}. Source data are provided as a Source Data file.

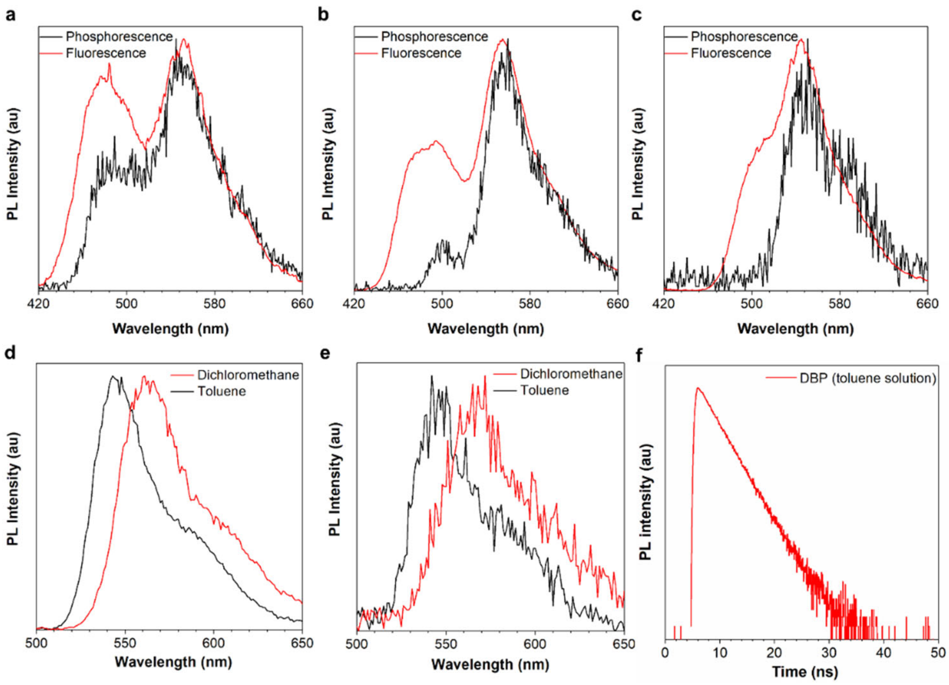

Supplementary Figure 8. Fluorescence and phosphorescence spectra of **a** film I, **b** film V and **c** film VI. **d** Fluorescence and **e** phosphorescence spectra of 4CzTPNBu in toluene and dichloromethane solutions measured at 77 K. **f** Transient PL decay curves of DBP in toluene solution. Source data are provided as a Source Data file.

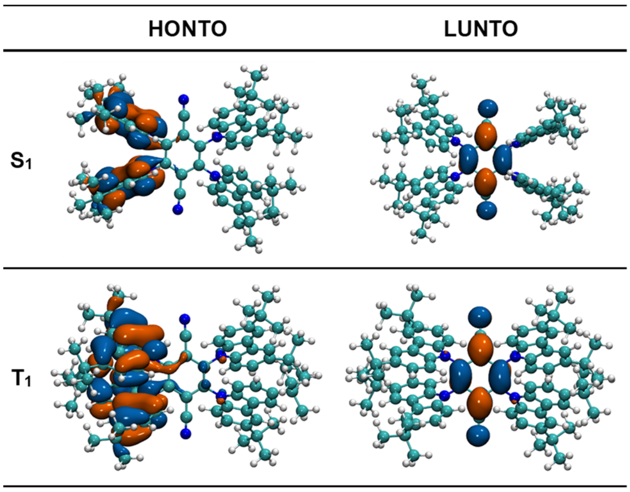

Supplementary Figure 9. Natural transition orbitals of singlet state and triplet state of 4CzTPNBu.

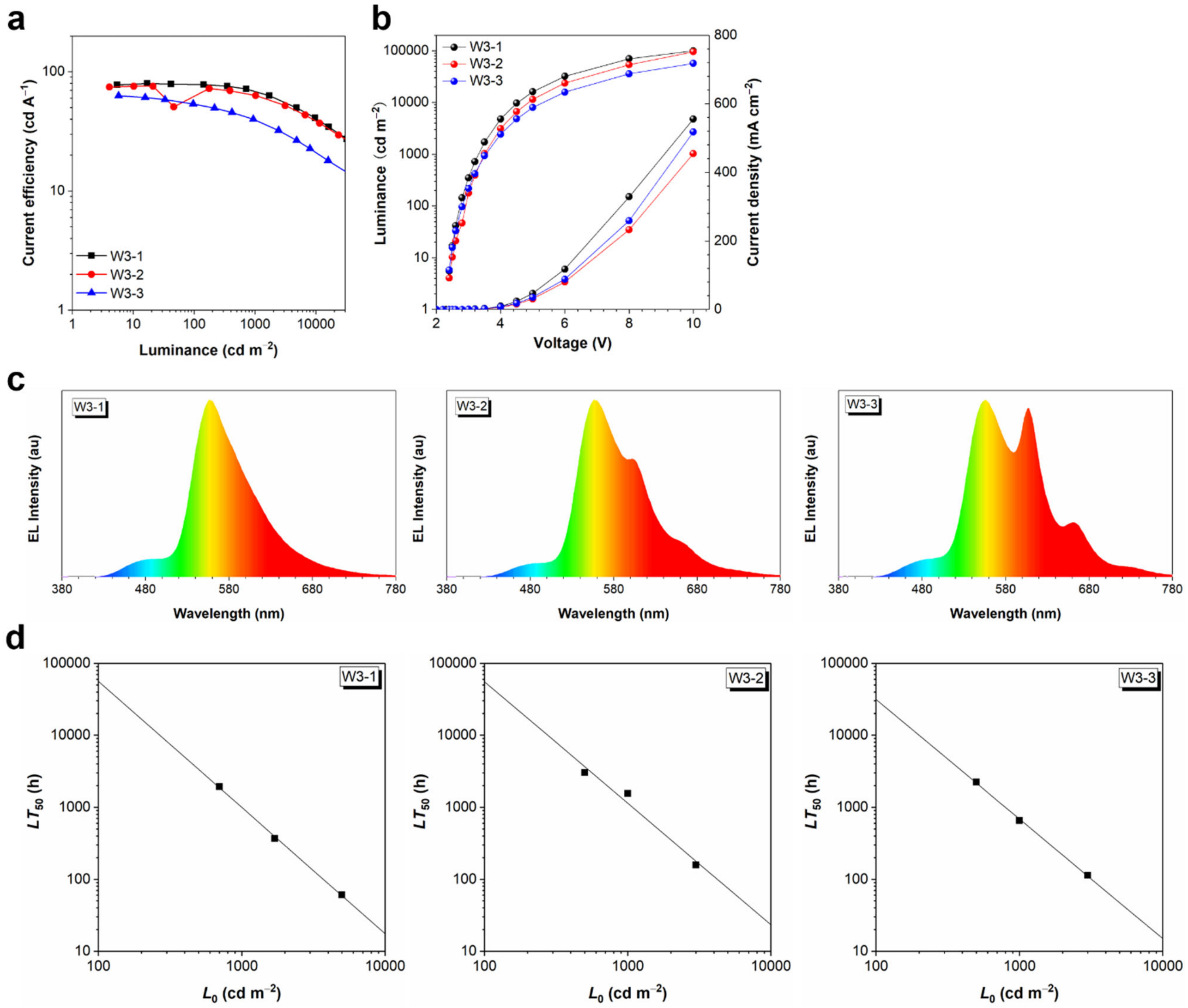

Supplementary Figure 10. **a** Current efficiency-luminance and **b** luminance-voltage-current density curves of devices W3-1~W3-3. **c** EL spectra of devices W3-1~W3-3 at ~1000 cd m^{-2}. **d** Relationships between L_0 and LT_{50} of devices W3-1~W3-3, where L_0 is the initial luminance. The operational lifetimes of the devices are fitted with formula

$L/L_0 = \exp\left(-\left(\frac{t}{\tau}\right)^{\beta}\right)$. The curves of L_0 $vs.$ LT_{50} are fitted from $L_0{}^{n}LT_{50} = constant$.[1] Source data are provided as a Source Data file.

Supplementary Table 1. Configurations of WOLEDs and films studied in this work.

Configurations of all WOLEDs	
**W1-0**	ITO/HATCN (5 nm)/TAPC (50 nm)/TcTa (5 nm)/mCP (5 nm)/TCP-BP-SFAC (15 nm)/1 wt% 4CzTPNBu: TCP-BP-SFAC (5 nm)/PPF (5 nm)/TmPyPB (40 nm)/LiF (1 nm)/Al
**W1-1**	ITO/HATCN (5 nm)/TAPC (50 nm)/TcTa (5 nm)/mCP (5 nm)/TCP-BP-SFAC (14 nm)/1 wt% 4CzTPNBu: TCP-BP-SFAC (5 nm)/TCP-BP-SFAC (1 nm)/PPF (5 nm)/TmPyPB (40 nm)/LiF (1 nm)/Al
**W1-2**	ITO/HATCN (5 nm)/TAPC (50 nm)/TcTa (5 nm)/mCP (5 nm)/TCP-BP-SFAC (13 nm)/1 wt% 4CzTPNBu: TCP-BP-SFAC (5 nm)/TCP-BP-SFAC (2 nm)/PPF (5 nm)/TmPyPB (40 nm)/LiF (1 nm)/Al
**W1-3**	ITO/HATCN (5 nm)/TAPC (50 nm)/TcTa (5 nm)/mCP (5 nm)/TCP-BP-SFAC (12 nm)/1 wt% 4CzTPNBu: TCP-BP-SFAC (5 nm)/TCP-BP-SFAC (3 nm)/PPF (5 nm)/TmPyPB (40 nm)/LiF (1 nm)/Al
**W1-4**	ITO/HATCN (5 nm)/TAPC (50 nm)/TcTa (5 nm)/mCP (5 nm)/TCP-BP-SFAC (11 nm)/1 wt% 4CzTPNBu: TCP-BP-SFAC (5 nm)/TCP-BP-SFAC (4 nm)/PPF (5 nm)/TmPyPB (40 nm)/LiF (1 nm)/Al
**W1-5**	ITO/HATCN (5 nm)/TAPC (50 nm)/TcTa (5 nm)/mCP (5 nm)/TCP-BP-SFAC (10 nm)/1 wt% 4CzTPNBu: TCP-BP-SFAC (5 nm)/TCP-BP-SFAC (5 nm)/PPF (5 nm)/TmPyPB (40 nm)/LiF (1 nm)/Al
**W1-6**	ITO/HATCN (5 nm)/TAPC (50 nm)/TcTa (5 nm)/mCP (5 nm)/TCP-BP-SFAC (9 nm)/1 wt% 4CzTPNBu: TCP-BP-SFAC (5 nm)/TCP-BP-SFAC (6 nm)/PPF (5 nm)/TmPyPB (40 nm)/LiF (1 nm)/Al
**W1-7**	ITO/HATCN (5 nm)/TAPC (50 nm)/TcTa (5 nm)/mCP (5 nm)/TCP-BP-SFAC (8 nm)/1 wt% 4CzTPNBu: TCP-BP-SFAC (5 nm)/TCP-BP-SFAC (7 nm)/PPF (5 nm)/TmPyPB (40 nm)/LiF (1 nm)/Al
**W1-8**	ITO/HATCN (5 nm)/TAPC (50 nm)/TcTa (5 nm)/mCP (5 nm)/TCP-BP-SFAC (7 nm)/1 wt% 4CzTPNBu: TCP-BP-SFAC (5 nm)/TCP-BP-SFAC (8 nm)/PPF (5 nm)/TmPyPB (40 nm)/LiF (1 nm)/Al
**W2-1**	ITO/HATCN (5 nm)/TAPC (50 nm)/TcTa (5 nm)/mCP (5 nm)/1 wt% DBP: TCP-BP-SFAC (9 nm)/1.5 wt% 4CzTPNBu: TCP-BP-SFAC (5 nm)/TCP-BP-SFAC (6 nm)/PPF (5 nm)/TmPyPB (40 nm)/LiF (1 nm)/Al
**W2-2**	ITO/HATCN (5 nm)/TAPC (50 nm)/TcTa (5 nm)/mCP (5 nm)/1 wt% DBP: TCP-BP-SFAC (10 nm)/1.5 wt% 4CzTPNBu: TCP-BP-SFAC (4 nm)/TCP-BP-SFAC (6 nm)/PPF (5 nm)/TmPyPB (40 nm)/LiF (1 nm)/Al
**W2-3**	ITO/HATCN (5 nm)/TAPC (50 nm)/TcTa (5 nm)/mCP (5 nm)/1 wt% DBP: TCP-BP-SFAC (11 nm)/1.5 wt% 4CzTPNBu: TCP-BP-SFAC (3 nm)/TCP-BP-SFAC (6 nm)/PPF (5 nm)/TmPyPB (40 nm)/LiF (1 nm)/Al
**W3-1**	ITO/MoO_3 (6 nm)/mCBP (40 nm)/1.5 wt% 4CzTPNBu: DIC-TRZ (14 nm)/20 wt% BCz-TRZ: TCP-BP-SFAC (6 nm)/TPBi (5 nm)/Bpy-TP2 (40 nm)/LiF (1 nm)/Al
**W3-2**	ITO/MoO_3 (6 nm)/mCBP (40 nm)/1 wt% DBP: DIC-TRZ (5 nm)/1.5 wt% 4CzTPNBu: DIC-TRZ (9 nm)/20 wt% BCz-TRZ: TCP-BP-SFAC (6 nm)/TPBi (5 nm)/Bpy-TP2 (40 nm)/LiF (1 nm)/Al
**W3-3**	ITO/MoO_3 (6 nm)/mCBP (40 nm)/1 wt% DBP: DIC-TRZ (8 nm)/1.5 wt% 4CzTPNBu: DIC-TRZ (6 nm)/20 wt% BCz-TRZ: TCP-BP-SFAC (6 nm)/TPBi (5 nm)/Bpy-TP2 (40 nm)/LiF (1 nm)/Al
**WS1**	ITO/HATCN (5 nm)/TAPC (50 nm)/TcTa (5 nm)/mCP (5 nm)/1.5 wt% 4CzTPNBu: TCP-BP-SFAC (5 nm)/TCP-BP-SFAC (6 nm)/PPF (5 nm)/TmPyPB (40 nm)/LiF (1 nm)/Al
**WS2**	ITO/HATCN (5 nm)/TAPC (50 nm)/TcTa (5 nm)/mCP (5 nm)/1.5 wt% 4CzTPNBu: TCP-BP-SFAC (14 nm)/TCP-BP-SFAC (6 nm)/PPF (5 nm)/TmPyPB (40 nm)/LiF (1 nm)/Al
**WS3**	ITO/HATCN (5 nm)/TAPC (50 nm)/TcTa (5 nm)/1 wt% DBP: mCP (5 nm)/1.5 wt% 4CzTPNBu: TCP-BP-SFAC (14 nm)/TCP-BP-SFAC (6 nm)/PPF (5 nm)/TmPyPB (40 nm)/LiF (1 nm)/Al

**WS4**	ITO/HATCN (5 nm)/TAPC (50 nm)/TcTa (5 nm)/mCP (5 nm)/1 wt% DBP: TCP-BP-SFAC (20 nm)/PPF (5 nm)/TmPyPB (40 nm)/LiF (1 nm)/Al
**WS5**	ITO/HATCN (5 nm)/TAPC (50 nm)/TcTa (5 nm)/mCP (5 nm)/1 wt% DBP: BDMAC-XT (9 nm)/1.5 wt% 4CzTPNBu: BDMAC-XT (5 nm)/TCP-BP-SFAC (6 nm)/PPF (5 nm)/TmPyPB (40 nm)/LiF (1 nm)/Al
**WS6**	ITO/HATCN (5 nm)/TAPC (50 nm)/TcTa (5 nm)/mCP (5 nm)/1 wt% DBP: BCz-TRZ (9 nm)/1.5 wt% 4CzTPNBu: BCz-TRZ (5 nm)/TCP-BP-SFAC (6 nm)/PPF (5 nm)/TmPyPB (40 nm)/LiF (1 nm)/Al
**WS7**	ITO/HATCN (5 nm)/TAPC (50 nm)/TcTa (5 nm)/mCP (5 nm)/BDMAC-XT (9 nm)/1.5 wt% 4CzTPNBu: TCP-BP-SFAC (5 nm)/TCP-BP-SFAC (6 nm)/PPF (5 nm)/TmPyPB (40 nm)/LiF (1 nm)/Al
**Y1**	ITO/HATCN (5 nm)/TAPC (50 nm)/TcTa (5 nm)/mCP (5 nm)/3 wt% 4CzTPNBu: TCP-BP-SFAC (20 nm)/PPF (5 nm)/TmPyPB (40 nm)/LiF (1 nm)/Al
**Y2**	ITO/HATCN (5 nm)/TAPC (50 nm)/TcTa (5 nm)/mCP (5 nm)/3 wt% 4CzTPNBu: BCz-TRZ (20 nm)/PPF (5 nm)/TmPyPB (40 nm)/LiF (1 nm)/Al
**Y3**	ITO/HATCN (5 nm)/TAPC (50 nm)/TcTa (5 nm)/mCP (5 nm)/3 wt% 4CzTPNBu: BDMAC-XT (20 nm)/PPF (5 nm)/TmPyPB (40 nm)/LiF (1 nm)/Al
**Configurations of all films**	
**Film I**	1.5 wt% 4CzTPNBu: TCP-BP-SFAC (15 nm)
**Film II**	1 wt% DBP: TCP-BP-SFAC (9 nm)/1.5 wt% 4CzTPNBu: TCP-BP-SFAC (5 nm)
**Film III**	1 wt% DBP: TCP-BP-SFAC (15 nm)
**Film IV**	TCP-BP-SFAC (15 nm)
**Film V**	1.5 wt% 4CzTPNBu: BCz-TRZ (15 nm)
**Film VI**	1.5 wt% 4CzTPNBu: BDMAC-XT (15 nm)

Supplementary Table 2. Comparison of EL performance of two-color WOLEDs.

[Ref]	Voltage (V)[a]	*PE* (lm W^{-1})[b]	*EQE* (%)[b]	CIE (x, y)[c]	L_{max} (cd m^{-2})[d]	Type
	V_{on}/100/1000 cd m^{-2}	Max/100/1000 cd m^{-2}	Max/100/1000 cd m^{-2}			
W1-6	2.5/2.8/3.2	130.0/109.9/78.5	31.1/29.7/25.3	(0.374, 0.505)	52690	Fluorescence
[2]	2.45/–/3.1	78.0/–/69.6	21.7/–/21.4	(0.399, 0.511)	>30000	Fluorescence
[3]	2.7/4.2/6.7	108.2/68.4/39.5	32.7/31.5/29.6	(0.44, 0.47)	37000	Fluorescence
[4]	2.6/~6.5/~7.0	99.9/~65/~50	32.8/28.4/24.1	(0.41, 0.46)	~70000	Fluorescence
[5]	2.5/2.9/3.6	105.0/82.5/59.5	28.1/24.0/21.5	(0.40, 0.48)	~20000	Phosphorescence
[6]	2.5/–/–	102.9/–/63.5	28.3/–/25.7	(0.45, 0.48)	~40000	Hybrid
[7]	2.3/–/3.4	92.0/–/72.7	28.2/–/28.0	(0.42, 0.48)	~30000	Phosphorescence

[a] Operating voltage at 1, 100 and 1000 cd m^{-2}; [b] *PE/EQE* = power efficiency/external quantum efficiency at maximum value, 100 and 1000 cd m^{-2}; [c] Commission Internationale de l'Eclairage coordinates at 1000 cd m^{-2}; [d] Maximum luminance.

Supplementary Table 3. EL performance of supplementary WOLEDs and monochromatic OLEDs.

	Voltage (V)[a]	*CE* (cd A^{-1})[b]	*PE* (lm W^{-1})[b]	*EQE* (%)[b]	CIE (x, y)[c]	L_{max} (cd m^{-2})[d]
	V_{on}/100/1000 cd m^{-2}	Max/100/1000 cd m^{-2}	Max/100/1000 cd m^{-2}	Max/100/1000 cd m^{-2}		
WS1	2.6/2.9/3.3	75.0/68.0/59.8	90.7/75.1/57.7	23.2/22.5/20.6	(0.324, 0.477)	57310
WS2	2.5/2.8/3.2	99.9/95.0/79.9	124.9/106.5/78.5	30.1/28.9/25.4	(0.400, 0.511)	82060
WS3	2.5/2.8/3.2	101.9/96.3/79.4	126.8/108.0/77.9	30.7/29.3/25.3	(0.394, 0.510)	82150
WS4	2.7/3.3/4.2	8.6/8.1/8.4	8.1/7.8/6.2	4.9/4.7/4.5	(0.406, 0.376)	15550
WS5	2.2/2.6/3.1	51.1/50.0/45.9	72.1/60.4/47.1	23.2/20.8/17.7	(0.503, 0.487)	90770
WS6	2.4/2.9/3.5	51.742.6/36.6	67.6/46.3/32.9	23.9/19.4/15.9	(0.447, 0.449)	41660
WS7	2.5/2.8/3.2	94.5/91.9/85.9	116.7/103.1/84.4	28.2/27.4/25.8	(0.410, 0.548)	88450
Y1	2.6/2.9/3.3	86.6/84.2/84.2	91.6/91.1/80.7	26.1/25.5/25.4	(0.451,0.535)	103200
Y2	2.5/2.9/3.3	73.8/73.2/72.7	87.1/81.2/69.6	22.6/22.4/22.2	(0.464,0.528)	141400
Y3	2.4/2.7/3.0	89.3/89.1/86.9	112.6/105.9/91.0	25.8/25.7/24.9	(0.433,0.556)	163900

[a] Operating voltage at 1, 100 and 1000 cd m^{-2}; [b] *CE*/*PE*/*EQE* = current efficiency/power efficiency/external quantum efficiency at maximum value, 100 and 1000 cd m^{-2}; [c] Commission Internationale de l'Eclairage coordinates at 1000 cd m^{-2}; [d] Maximum luminance.

Supplementary Table 4. Comparison of EL performance of three-color WOLEDs.

[Ref]	Voltage (V)[a]	*PE* (lm W^{-1})[b]	*EQE* (%)[b]	CIE (x, y)[c]	L_{max} (cd m^{-2})[d]	Type
	V_{on}/100/1000 cd m^{-2}	Max/100/1000 cd m^{-2}	Max/100/1000 cd m^{-2}			
W2-1	2.5/2.9/3.4	106.8/80.6/52.6	30.8/26.4/20.6	(0.394, 0.476)	42410	Fluorescence
[8]	3.4/-/5.4	51.4/-/21.8	19.2/-/14.2	(0.348, 0.457)	~20000	Fluorescence
[2]	2.45/-/-	43.3/-/39.8	16.7/-/16.5	(0.439, 0.452)	>30000	Fluorescence
[9]	2.5/3.4/4.8	84.1/48.7/24.2	25.5/20.2/14.1	(0.40, 0.43)	~10000	Hybrid
[10]	2.7/~4/~5.5	48.3/34.5/24.1	25.6/25.3/25.1	(0.41, 0.46)	25540	Hybrid
[11]	2.4/~3.0/~4.0	89.0/60.0/51.1	22.9/22.6/21.7	(0.436, 0.421)	>50000	Phosphorescence

[a] Operating voltage at 1, 100 and 1000 cd m^{-2}; [b] *PE/EQE* = power efficiency/external quantum efficiency at maximum value, 100 and 1000 cd m^{-2}; [c] Commission Internationale de l'Eclairage coordinates at 1000 cd m^{-2}; [d] Maximum luminance.

Supplementary Table 5. Transient PL decay data of films I~IV recorded at various PL wavelengths.

	τ_{prompt} (ns)[a]	$\tau_{delayed}$ (μs)[b]	R_{prompt} (%)[c]	$R_{delayed}$ (%)[d]
Film I-480 nm	22.5	2.19	57.1	42.9
Film I-550 nm	31.0	2.52	29.8	70.2
Film II-480 nm	25.6	1.72	66.8	33.2
Film II-550 nm	30.5	1.99	45.7	54.3
Film II-610 nm	24.2	1.55	69.4	30.6
Film III-480 nm	22.3	1.33	71.8	28.2
Film III-610 nm	20.9	1.02	81.1	18.9
Film IV-480 nm	25.7	3.02	65.2	34.8
Film V-560 nm	20.5	1.79	80.1	19.9
Film VI-550 nm	22.3	1.47	75.4	24.6

[a] Prompt fluorescence lifetime; [b] Delayed fluorescence lifetime; [c] Ratio of prompt component; [d] Ratio of delayed component.

Supplementary Table 6. Calculated molecular polarity index (MPI) of S_1 and T_1 states of TCP-BP-SFAC, BCz-TRZ and BDMAC-XT.

Host	MPI of S_1 (kcal mol^{-1})	MPI of T_1 (kcal mol^{-1})
TCP-BP-SFAC	14.54	8.72
BCz-TRZ	22.55	10.78
BDMAC-XT	12.00	9.98

Supplementary References

1. Féry, C., Racine, B., Vaufrey, D., Doyeux, H., Cinà, S. Physical mechanism responsible for the stretched exponential decay behavior of aging organic light-emitting diodes. *Appl. Phys. Lett.* **87**, 213502(2005).
2. Zhang, C., Lu, Y., Liu, Z., Zhang, Y., Wang, X., Zhang, D., & Duan, L. A π–D and π–A Exciplex-Forming Host for High-Efficiency and Long-Lifetime Single-Emissive-Layer Fluorescent White Organic Light-Emitting Diodes. *Adv. Mater.* **32**, 2004040(2020).
3. Han, C., Du, R., Xu, H., Han, S., Ma, P., Bian, J., Duan, C., Wei, Y., Sun, M., Liu, X., Huang, W. Ladder-like energy-relaying exciplex enables 100% internal quantum efficiency of white TADF-based diodes in a single emissive layer. *Nat. Commun.* **12**, 3640(2021).
4. Chen, J. X., Wang, K., Xiao, Y. F., Cao, C., Tan, J. H., Wang, H., Fan, X.-C., Yu, J., Geng, F.-X., Zhang, X.-H., Lee, C. S. Thermally Activated Delayed Fluorescence Warm White Organic Light Emitting Devices with External Quantum Efficiencies Over 30%. *Adv. Funct. Mater.* **31**, 2101647(2021).
5. Wu, S. F., Li, S. H., Wang, Y. K., Huang, C. C., Sun, Q., Liang, J. J., Liao, L.-S., Fung, M. K. White Organic LED with a Luminous Efficacy Exceeding 100 lm W^{-1} without Light Out-Coupling Enhancement Techniques. *Adv. Funct. Mater.* **27**, 1701314(2017).
6. Wu, Z., Yu, L., Zhao, F., Qiao, X., Chen, J., Ni, F., Yang, C., Ahamad, T., Alshehri, S. M., Ma, D. Precise exciton allocation for highly efficient white organic light-emitting diodes with low efficiency roll-off based on blue thermally activated delayed fluorescent exciplex emission. *Adv. Optical Mater.* **5**, 1700415(2017).
7. Tang, X., Liu, X.-Y., Yuan, Y., Wang, Y.-J., Li, H. C., Jiang, Z.-Q., Liao, L.-S. High-efficiency white organic light-emitting diodes integrating gradient exciplex allocation system and novel D-spiro-A materials. *ACS Appl. Mater. Interfaces* **10**, 29840-29847(2018).
8. Li, X. L., Xie, G., Liu, M., Chen, D., Cai, X., Peng, J., Cao, Y., Su, S.-J. High-efficiency WOLEDs with high color-rendering index based on a chromaticity-adjustable yellow thermally activated delayed fluorescence emitter. *Adv. Mater.* **28**, 4614-4619(2016).
9. Liu, X. K., Chen, Z., Qing, J., Zhang, W. J., Wu, B., Tam, H. L., Zhu, F., Zhang, X.-H., Lee, C.-S. Remanagement of singlet and triplet excitons in single-emissive-layer hybrid white organic light-emitting devices using thermally activated delayed fluorescent blue exciplex. *Adv. Mater.* **27**, 7079-7085(2015).
10. Liang, J., Li, C., Zhuang, X., Ye, K., Liu, Y., Wang, Y. Novel Blue Bipolar Thermally Activated Delayed Fluorescence Material as Host Emitter for High-Efficiency Hybrid Warm-White OLEDs with Stable High Color-Rendering Index. *Adv. Funct. Mater.* **28**, 1707002(2018).
11. Ying, S., Yuan, J., Zhang, S., Sun, Q., Dai, Y., Qiao, X., Yang, D., Chen, J., Ma, D. High efficiency warm white organic light-emitting diodes with precise confinement of charge carriers and excitons in the exciplex host system. *J. Mater. Chem. C* **7**, 7114-7120(2019).
